# Global Patterns in the Implementation of Payments for Environmental Services

**DOI:** 10.1371/journal.pone.0149847

**Published:** 2016-03-03

**Authors:** Driss Ezzine-de-Blas, Sven Wunder, Manuel Ruiz-Pérez, Rocio del Pilar Moreno-Sanchez

**Affiliations:** 1 CIRAD (Centre de Coopération Internationale en Recherche Agronomique pour le Développement), Montpellier, France; 2 CIFOR (Center for International Forestry Research), Lima, Peru; 3 UAM (Autonomous University of Madrid), Madrid, Spain; 4 Conservation Strategy Fund, Bogota, Colombia; Universitat Jaume I, SPAIN

## Abstract

Assessing global tendencies and impacts of conditional payments for environmental services (PES) programs is challenging because of their heterogeneity, and scarcity of comparative studies. This meta-study systematizes 55 PES schemes worldwide in a quantitative database. Using categorical principal component analysis to highlight clustering patterns, we reconfirm frequently hypothesized differences between public and private PES schemes, but also identify diverging patterns between commercial and non-commercial private PES vis-à-vis their service focus, area size, and market orientation. When do these PES schemes likely achieve significant environmental additionality? Using binary logistical regression, we find additionality to be positively influenced by three theoretically recommended PES ‘best design’ features: spatial targeting, payment differentiation, and strong conditionality, alongside some contextual controls (activity paid for and implementation time elapsed). Our results thus stress the preeminence of customized design over operational characteristics when assessing what determines the outcomes of PES implementation.

## Introduction

Payments for environmental services (PES) have become an increasingly popular tool for environmental management, supplementing policy tools that were previously widely focused on command-and-control measures. Dozens of programs to reward provision of environmental services (ES) are currently being implemented at multiple geographical scales around the world See e.g. www.watershedconnect.com, www.ecosystemarketplace.com and http://www.oas.org/dsd/PES/Database.htm#). PES feature direct, conditional contracts to achieve a negotiated environmental outcome between a provider and a user of environmental services [1]. The underlying rationale is that if the service user’s gain from pro-environmental action is sufficiently large, there may be a good case for compensating the service-providing landowners for choosing a profit-wise second-best, but environmentally more benign resource use. This type of “Coasean deal” implies using a positive economic incentive to enhance a positive (or avoid a negative) environmental externality by internalising the costs of conservation [1, 2, 3]. PES theory distinguishes the tool from other positive conservation incentives (e.g. integrated conservation and development programs, certification) by the notion of direct, conditional ‘quid pro quo’ payments–i.e. user payments are to be withdrawn when it can be verified that providers did not comply with their assumed obligations [1, 2]. Conditionality is a core defining characteristic for PES, in the way we are interpreting and delimiting the concept in the following [4,5].

The popularity of PES also relates to a historical moment of economic and environmental thought [6]. Much academic interest has been dedicated to disentangling the concept and principles of PES [7–11] and how their design features link to socio-environmental impacts [12–16] and trade-offs [17–19]. Hence, important research questions arise from this literature. For instance, for which targeted environmental services and scales are user-financed PES schemes more likely to emerge than government-financed ones? When are the former likely to be more efficient than the latter, and *vice versa*? In health, banking, transport and water provision, the comparative additionality of private vs. public sectors has been debated much more thoroughly than for environmental services [20–22]. Particularly, so far we widely lack robust quantitative analysis of global patterns linking targeted ES, geographic location, involved actors, design features, and environmental additionality. Understanding such empirical patterns emerging from the growing body of case studies worldwide could help us gain new insights for policies and best practices [23].

Notably, private and public sector PES implementation models emerge, co-exist and cooperate worldwide. Private PES are usually negotiated and customized to local conditions, including so that ES buyers can directly sanction any non-compliance by ES providers [23]. The Profafor carbon PES in Ecuador [24], the Vittel watershed scheme in France [25] and the Simanjiro wildlife conservation scheme in Tanzania [26] are such examples of private PES. In publicly financed PES, local or national governments act to congregate ES user interests by levying taxes or fees on end users or tax payers -thus remedying for free-rider problems- and earmarking revenues for conditional payments to ES providers [27]. The Sloping Land Conversion Program in China [28], the Conservation Reserve Program in the USA [29], the PES national program in Costa Rica [30], and the Payments for Hydrological Services Program in Mexico [31] are prominent examples. However, public-private PES hybrids with sequential sector leadership are also found sometimes [32,33]. Here we choose the financing criterion to distinguish public from private schemes; other studies featured the proximity and influence of ES users as key criteria of PES categorization [2]. Hence, some municipal local-scale PES (e.g. watershed schemes) there classified as user-financed in our sample become publicly financed PES schemes, because a public sector entity acts as collector and custodian of PES funding.

To our knowledge, only three quantitative-comparative PES studies pre-exist. First, [34] analysed 47 watershed PES schemes worldwide. They confirmed a positive influence of directness (i.e. absence of intermediaries) on environmental effectiveness, as self-perceived by project implementers. Second, [35] had 22 broadly classified PES initiatives in Germany and the USA assessed by 26 socio-environmental experts for their perceived socio-environmental success. Voluntariness, government participation, and long-term contracts were all found to be associated with higher frequency of success in their small two-country sample of PES and PES-like schemes. Third, [36] studied how natural, financial, institutional and socio-economic capital varied across 23 PES schemes in developing and emergent tropical countries. They found that PES improved both natural and social capital stocks, and that institutional arrangements fall in two categories: state-structured and privately financed.

In the present study we aim to strengthen the global-comparative empirical basis of PES functionality in various respects, by conducting a global-scale meta-analysis. In particular, we look at the interplay between PES implementation characteristics, contextual and institutional factors (including the role of the public and private sectors), and evidence of environmental additionality. There has been extensive debate whether PES should be broadly defined, or follow a narrower, theoretically-based definition [2,5,8,10]. In the following, we choose a narrower delimitation, focused on conditionality as *sine qua non* PES criterion [4]. While this choice evidently caps our sample size, it allows us to look at interventions that are truly comparable. We refer to schemes fitting all five criteria as “canonical PES”, and include in our sample only schemes that deviate moderately from this ideal setup (see below). We also only include cases with one or more pre-existing academic assessments in the literature, aiming thus at assessing only PES schemes with scientifically validated sources of information.

By adopting a globally scoped systematic literature review, including all terrestrial environmental services, we reach a sample of 55 PES schemes. Compared to [34] and [35], we extend the set of design-oriented (e.g. type of payments, use of baselines, monitoring) and context-specific variables (e.g. size, region, services transacted, actors involved) in our statistical analysis. Compared to [36], our twice as large sample enhances the statistical analysis, and treats new research questions. We thus believe our sample is as broad as the current state of PES affairs permits, and yet generically similar to be able to compare initiatives.

This article is structured as follows. In Section 2, we describe in detail our sample and methods. In Section 3 (Results), we follow a sequential approach, going from open-ended exploratory methods to an analysis with an assumed causal direction. We start with (a) descriptive statistics, then turn to (b) a categorical principal component analysis of PES variables, and lastly (c) analyse econometrically how PES design likely affects environmental additionality. Finally, we discuss the scope of our findings, and their implications for future research and policies (Section 4).

## Sample and Methods

While hundreds of PES schemes are reported loosely upon in the literature, most contributions do not provide sufficient in-depth information to be useful for quantitative meta-analysis. We selected our case studies based on different methodological guidelines for meta-analysis, derived from clinical and social sciences [37–39]. We carried out a systematic literature research drawing on various pre-existing PES databases, qualitative PES reviews, journal storage (JSTOR) and other web-based (Google Scholar) sources (Table 1). From the resulting identified records, we filtered out cases where payments to ES providers (i) could not be confirmed to have occurred at least once (never mind whether the program was still ongoing); (ii) did not conform well to our conditionality-focused definition for PES; or (iii) did not in the PES case study deliver sufficient descriptors to meet our minimum data standards.

**Table 1:** Database search protocol for PES studies included in study sample.

Database	Search strategy	Search terms	Total references (after duplicates)	Filtering conditions	Total meta-analysis references
Science direct	**Databases**: All sources**Subjects included**: All search terms**Searched in**: Title, abstracts and keywords**Dates**: 2000–2014	Payments for ecosystem services, OR payments for environmental services OR, singly or linked to the following: *additionality, *ecosystem services, *assessment, *public sector, *private sector, *biodiversity, *watershed services, *Asia, *Europe, *USA, *Latin America, *South America,*[Country name]	276 (157)	Selected studies contain: (i) a scheme that respects conditionality definition; AND (ii) has at least made one cycle of payments; AND (iii) offers detailed PES implementation of case study description AND (ii) quantitative OR qualitative scientific evidence on the scheme environmental additionality	33
Scopus	**Databases** (content sources): All sources**Document type**: All search terms**Searched in**: Article title, abstract, keywords.**Dates**: 2000–2014		493 (338)		48
German national library https://portal.dnb.de	**Search in**: Online Catalogue**Search**: All search terms		16 (15)		1
Open Greyhttp://www.opengrey.eu/	**Search**: All search terms		10 (10)		1
HighWirehttp://highwire.stanford.edu	**Search Title and Abstract only**: All search terms**Databases**: HighWire-hosted**Dates**: 2000 –Present		24 (20)		3
British national libraryhttp://www.bldss.bl.uk	**Search**: All search terms		250 (25)		1
Google Scholar	**Search**: All search terms		500* (3)		3
Brazilian scientific electronic library onlinehttp://www.scielo.br/	**Search**: All search terms**In field**: All indexes		2 (2)		0

**Fig 1:**
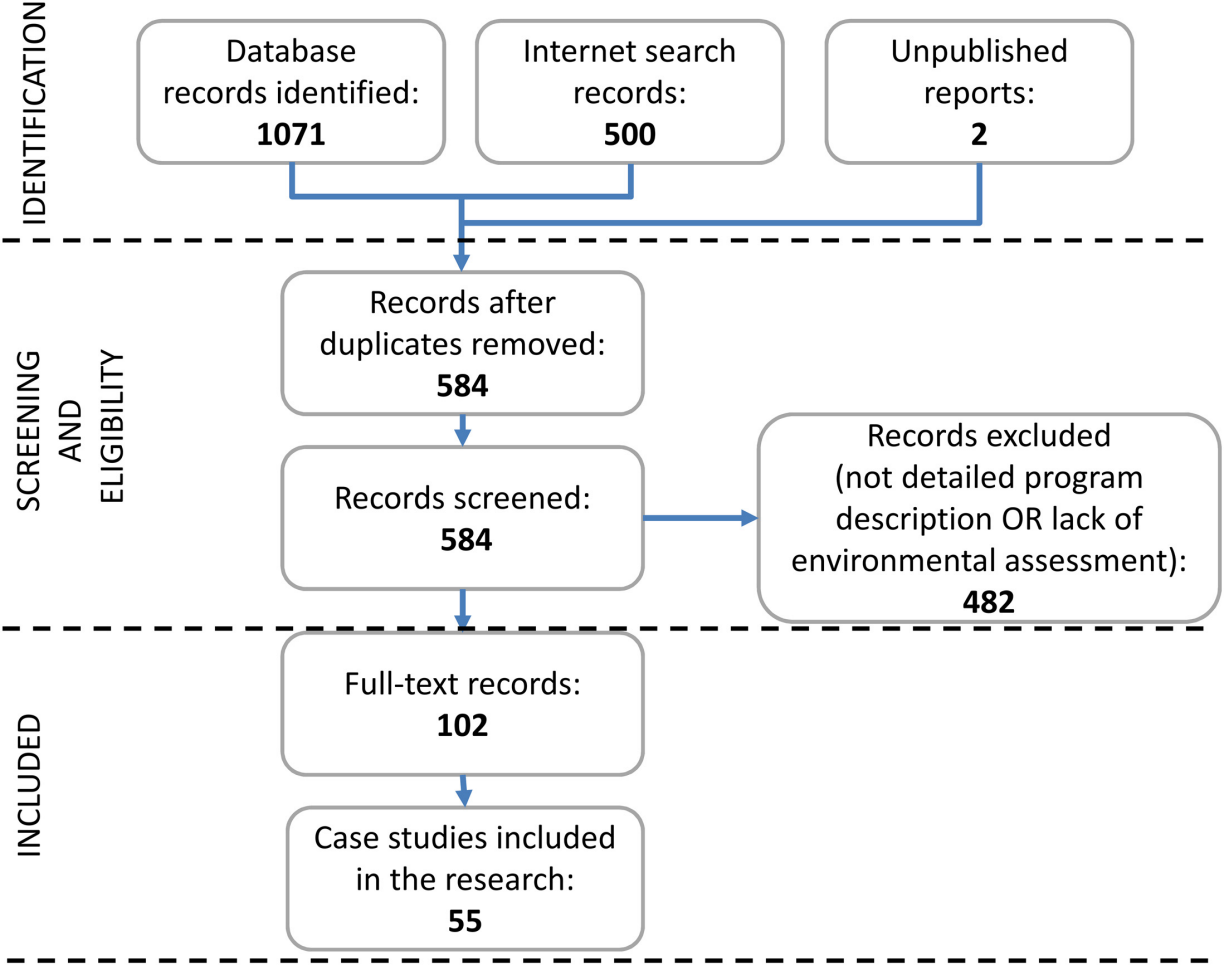
PRISMA flowchart for the identification and selection of PES schemes included in the study.

**Fig 2:**
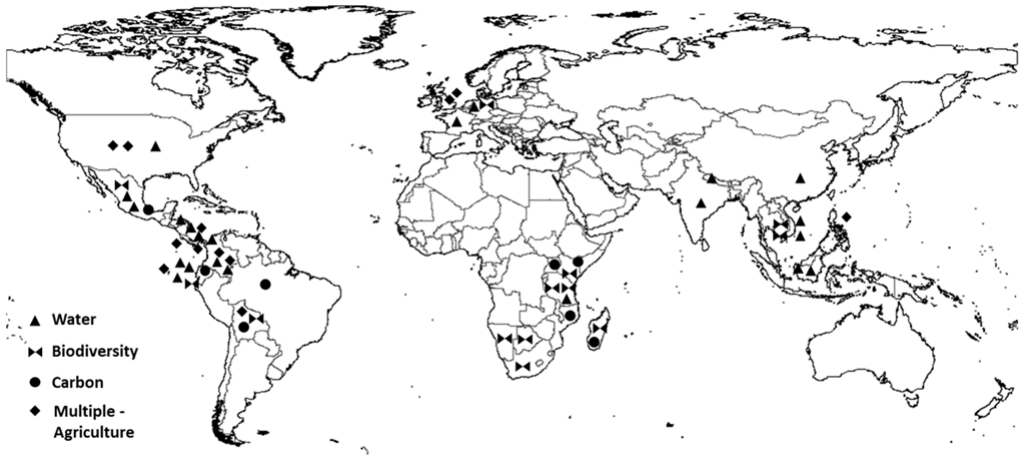
Location of PES schemes analysed.

This narrowed our sample to 90 literature references referring to a total of 55 PES cases worldwide (counting until mid-2014 when our statistical analysis was begun), of which 47 are ongoing (See S1 Table for meta-analysis references and Fig 1 for the PRISMA selection diagram). Fig 2 shows the geographic and thematic focus of the selected PES cases. They feature payments for the protection and restoration of watersheds (Water) (with 22 cases, the most frequently targeted ES), for biodiversity conservation (Biodiversity) (10 cases), for climate change mitigation through carbon sequestration or avoided deforestation (Carbon) (8 cases), and for multiple services from agriculturally dominated systems (Multiple-Agriculture) (12 cases). Region-wise, countries with emerging economies dominate, especially Latin America with 23 cases. Industrialised countries have less PES cases, though some are huge government schemes that outsize small-scale initiatives by orders of magnitude. We did not weight our observation in terms of scheme size, but treated each case equally. Hence, our analysis comes to put relatively more emphasis on a series of smaller-scale PES schemes that predominate in non-OECD countries.

We built a database of in total 50 basic variables, a dozen of which we employ analytically. The main categories are:

1. implementation modes (broad PES scheme descriptors, land-ES link, spatial extent);
2. program design (monitoring, sanctioning, baselines, ES targeting, differentiation of payments);
3. program institutional arrangements (ES buyer and provider types, source of payments, presence of intermediaries, transaction setting);
4. program funding (degree of user-financing, length of the contract, type and level of payment);
5. scheme adherence to our PES “canonical” standards (indexed degree of fit with PES theory); and
6. evidence of environmental additionality (unit measured, method, level).

Generally, we combined categorical (no hierarchy between levels), ordinal (hierarchical between levels), and continuous variables (cardinal ranking) in our analysis. S1 Database describes all variables in detail, but a few points are worth flagging. We registered which actors participated in different implementation stages, and classified them between public, private commercial (enterprises) and private non-commercial (NGOs, foundations, grassroots organizations) sectors (iii). Under funding sources (iv), we describe which sectors assumed which payments (cash and in-kind). Costs were divided into upfront (e.g. information, design, capacity building and negotiation costs) versus recurrent management costs (e.g. administrative, implementation, monitoring costs), and payments proper. Schemes totally or mainly funded by the public sector were classified as public-sector schemes; correspondingly for the two other sectors.

Fitness to a “canonical PES” (v) is a composite indicator derived from the sum of the criteria that refer to the compliance or not with the five PES definitional criteria mentioned above, plus the degree of transfer directness (adapted from [8]). This composite indicator allows us to capture the degree of implementation closeness to PES theory, using an ordinal indicator with scores ranging from 6 to 17 (S2 Table). The variable “economic setting” differentiates between degrees of competition on both provider and user sides (e.g. monopsony, oligopsony, club, market) [35]. Descriptive statistics of the main variables are shown in Table 2.

**Table 2:** Descriptive statistics for our main variables.

Variable	Type	Unit	Mean	SD	Median	Min.	Max.	Levels
PES objective	Cat	-	2,18	1,19	2.0	1	4	1 = Watershed protection; 2 = Biodiversity protection; 3 = Climate change mitigation; 4 = Multiple
Ecosyst. type	Cat	-	1,65	0,7	2.0	1	3	1 = Forest; 2 = Farmland; 3 = Semi-arid grasslands
Log10 size	Quant	Log10(ha)	3.99	1.72	3.9	0.90	7.20	
Sector	Cat	-	2.29	0.87	3.0	1	3	1 = Private; 2 = Non-profit; 3 = Government
Transaction costs	Cat	-	1.44	0.69	1.0	1	3	1 = Public; 2 = Non-profi;t 3 = Private
Running costs	Cat	-	1.65	0.82	1.0	1	3	1 = Public; 2 = Non-profit; 3 = Private
Payment costs	Cat	-	1.73	0.89	1.0	1	3	1 = Public; 2 = Non-profit; 3 = Private
Market setting	Cat	-	1.75	0.98	1.0	1	3	1 = Monopsone; 2 = Oligopsone; 3 = Club; 4 = Market
Payers are users	Ord	-	1.53	0.50	2.0	1	2	1 = No; 2 = Yes
Conditionality (Monitoring*Sanction)	Ord	-	3.96	2.41	3.0	1	9	
Fitness to Coasean definition	Ord	-	12.22	2.24	12,0	8	17	
Log10 payment per ha	Quant	Log10(USD/ha)	1.72	1.01	1.9	-0.68	3.58	
Additionality	Ord	-	0.76	0,43	1.0	0	1	0 = No significant; 1 = Significant
Activity paid	Cat.	-	0.60	0.49	1.0	0	1	0 = Conservation;1 = Asset-building PES
Diversification of payments	Cat.	-	0.65	0.48	1.0	0	1	0 = No; 1 = Yes
Spatial targeting	Cat.	-	1.05	0.65	1.0	0	2	0 = No targeting; 1 = Threat or ES density; 2 = Both
Additionality precision	Ord.	-	3.00	1.37	3,0	1	5	1 = Weak; 2 = Fragile; 3 = Medium; 4 = Strong; 5 = Rigurous

Finally, environmental additionality (vi) was clearly the most challenging variable, but also of great interest [40]. One previous meta-analysis used expert scores [35], another implementer self-assessment [34] of the degree of environmental success. Potentially we could have focused exclusively on studies using so-called rigorous impact assessment methods. Due to the scarcity of those so far in assessing PES [41], a recent systematic PES literature review applying that filter ended up with just 11 studies, six of which alone on Costa Rica’s PSA program [42]. Paradoxically, even among those six “rigorous” studies on Costa Rica, non-trivial differences exist in their bottom-line additionality, including because of variability in the methods used (e.g. matching techniques and controlling for sources of impact heterogeneity).

Even when researchers have not yet produced the perfect data sets for quantitative impact evaluation, practitioners and policy makers still need to make real-world choices about how to design their interventions, taking stock of the current state of affairs in the best possible way. In this study, we have thus opted for including both quantitative and qualitative documentation on environmental additionality, and classified it according to the precision of the assessment methods, resulting in five levels (S3 and S4 Tables). Consequently, we opted for the least pretentious outcome assessment: a binary variable to differentiate between, on the one hand, likely zero or low and on the other, likely considerable additionality in ES delivery and/or a targeted land-use proxy, such as forest cover. In addition, we performed various sensitivity analyses on our additionality assessments. From the total of 55 PES cases, for four simply no attempt had been made to gauge environmental additionality, thus restraining the number of cases for the additionality analysis to 51.

## Results

### Descriptive statistics

Our first interest is whether, as hypothesized in the PES literature, substantial differences exist between the characteristics of public vs. privately funded PES schemes [2,17]. Geographically, sector funding exhibits three patterns: In Latin America, about one tenth of cases are private non-commercial initiatives (9%), one fourth is private commercial, while two thirds (65%) are publicly funded cases. Europe, North America and Asia present a slightly higher frequency of publicly funded PES (70%). In Africa, privately funded schemes clearly predominate (85%). Half of these private schemes are run by the private commercial sector, especially for eco-tourism and wildlife. Do sectors alternate their lead of PES schemes over time? For one fourth of our cases, this is the case. The sector initiating the PES scheme (carrying upfront costs) also continues covering the recurrent management costs in 73% of cases. Where shifts occur, most frequently the public sector covers the initial costs, transferring the project afterwards to the private commercial sector to manage it. For instance, the Scolel Té project in Southern Mexico had initial funding from the UK Department for International Development (DFID) in partnership with the Mexican university *El Colegio de la Frontera Sur* (Ecosur) and the University of Edinburgh to kick-start this forest carbon scheme, while currently it is managed by the private company Ambio [43]. Such sequential transfer of leadership between private and public environment sectors has also been observed in other sectors [22].

**Fig 3:**
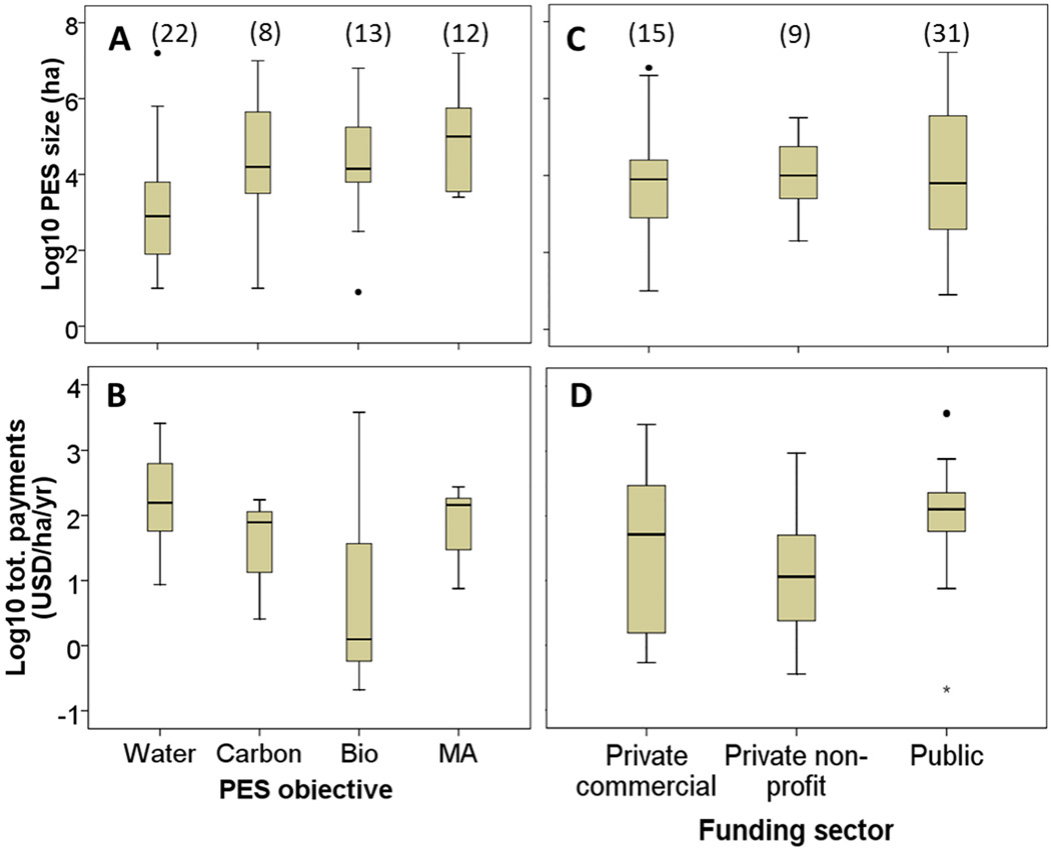
Box plot showing the distribution—median, interquartile range (box), upper and lower values below 1.5 interquartile range and outliers, of total cash payments and PES area size in a logarithmic (base 10) scale by ES targeted (sub-Figs A and B) and economic sector (C and D). In parenthesis are number of observation for each category.

How do per-hectare payments vary across the sample? Our oldest PES scheme, the Conservation Reserve Program (USA), started in 1985. Hence, we adjusted payments for inflation, converting to real US$ from 2003 –the median starting year of assessed PES cases (Source: http://www.oecd-ilibrary.org/statistics). Mean payments and size of PES schemes are both significantly different across ES targeted (ANOVA F = 9.5 p = 0.00 for payments, ANOVA F = 3.6 p = 0.02, for area). Watershed PES schemes are the smallest in size (Fig 3A), but record the highest annual per-hectare payments (3B), compared to other ES. Carbon, biodiversity and multi-functional agriculture PES schemes score similar in size, although carbon PES show large variability. Multifunctional agricultural PES schemes vary the least in payment amounts, and account for large areas. The lowest payments are for biodiversity PES, but with large variance. Unsurprisingly, public schemes are on average larger in size than private ones, especially vis-à-vis non-commercials, but also pay more per hectare (3C, 3D). As expected, payments vary much less in public than in private schemes (see S5 Table for details)

### Variable patterns and clusters

**Fig 4:**
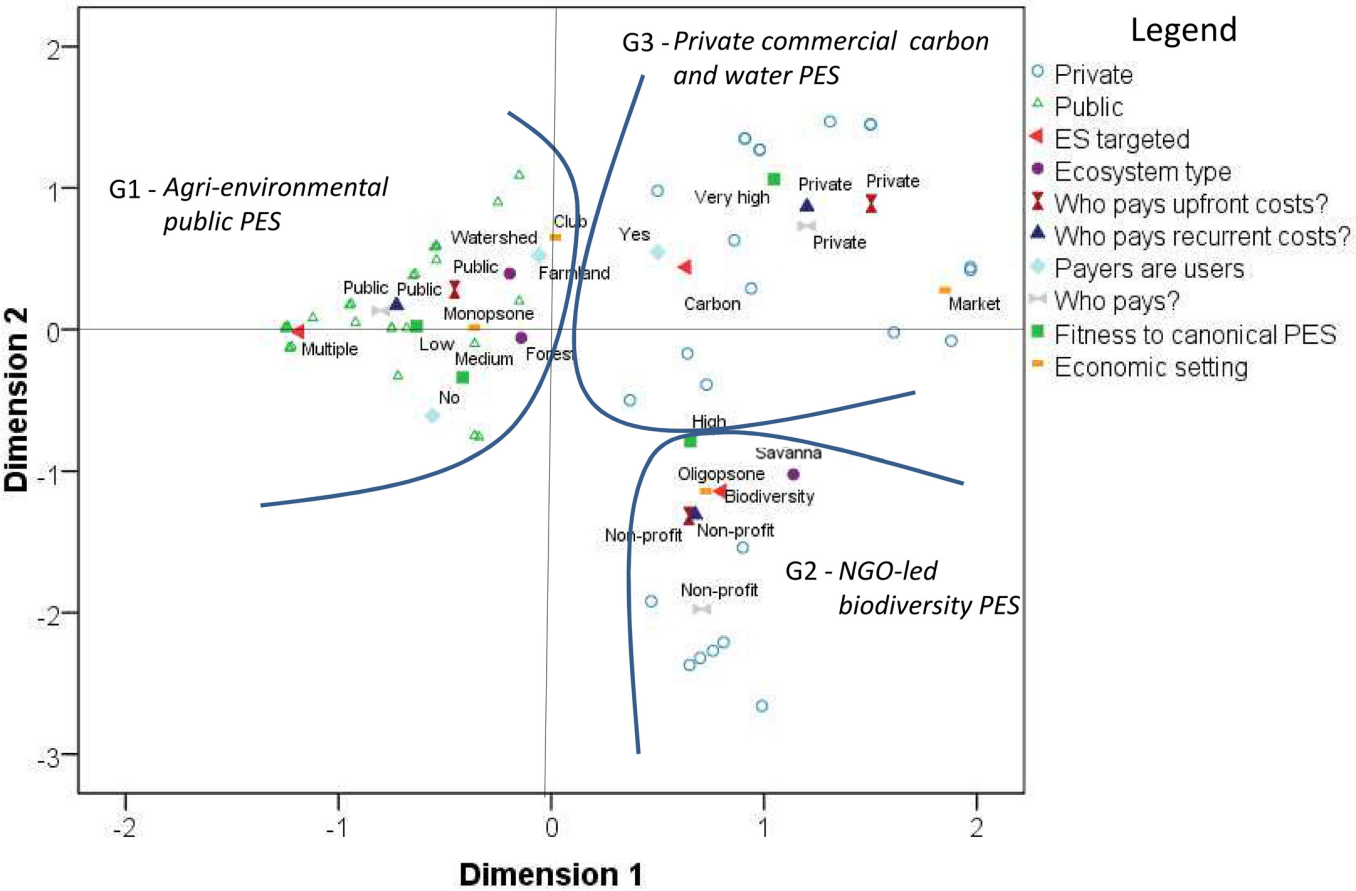
Categorical principal component analysis of main PES design types (see also S2 Table). Triangles refer to public PES, circles to private. G1, G2, G3 refer to PES schemes belonging to cluster groupings 1, 2, and 3, respectively.

PES schemes typically exhibit differences across services and implementing sectors (public vs. private), but do characteristics cluster in more systematic ways, as hypothesized in the PES literature [4,20]? In this subsection, we use multi-dimensional categorical principal component (CatPCA) and cluster analyses to explore underlying patterns. The analyses were conducted in a matrix of 55 PES (cases) and eight relevant categorical variables (attributes), including those synthetizing PES categories as defined by [35], and the composite index describing the “canonical PES” fitness. CatPCA reduces the multidimensional space represented by this matrix into a given number (usually two or three) of orthogonal (independent) dimensions. Each dimension is defined through a combination of the variables, and represents in decreasing order a given percentage of the total variability in the matrix. The data in the matrix have a strong internal consistency and reliability (Cronbach's Alpha = 0.87 –S6 Table). Fig 4 shows the first two dimensions of the analysis, representing a 52.1% of the total variability of our 55 cases per 8 variables matrix.

We additionally performed a cluster analysis (distance measure: Sorensen; group linkage method: group average) resulting in three distinct groupings of cases and attributes. The drawn border lines in Fig 4 delimit the PES schemes belonging to each of the three clusters. The combined CatPCA and cluster show which schemes have closest coordinates, and thus are more similar, the degree of concentration of PES schemes within groups, and the distance between groups. Variables with extreme values in one or both of the two axes define the cluster characteristics, since each axis is a vector composed by the weights of all the variables. On the contrary, variables close to the origin of the quadrant lines are variables that are present in two or more groups, and therefore do not explain the differences between groups. We identify the following three groupings:

- Group 1 (G1) has typical “*agri-environmental public PES*” characteristics, being defined by the association of: farmland ecosystems, multi-functional agricultural ES, public sector carrying upfront, management and payment costs, monopsony setting, low-to-medium scores for the fitness to a “canonical PES”, source of payments is not ES users;
- Group 2 (G2) could be called “*NGO-led biodiversity PES*”, as it is defined by the association of: semi-arid ecosystems, biodiversity as main ES target, non-profit sector carrying upfront, management and payment costs, oligopsony economic setting, high score for the fitness to a “canonical PES”;
- Group 3 (G3) we labeled “*Private commercial carbon and water PES*”, being defined by the association of: PES schemes targeting carbon-related ES, private sector carrying upfront, management and payment costs, high to very-high scores with regard to canonical PES index, source of payments is ES users.

While the PES literature previously had recognized important differences between public and private PES schemes [9,17], our analysis here thus also points to important differences within the private group between commercial and NGO-led PES schemes, e.g. with respect to ES focus and financing sources: a threefold sectoral categorization is thus empirically more justified. Conversely, it is also interesting to observe that forest ecosystems are placed very close to the intersection of both axis, suggesting that conserving forested ecosystems through PES is being approached from a combination of agri-environmental, carbon and biodiversity schemes.

c) PES design and the degree of additionality

In almost any PES literature review, a key reader question will be “what schemes worked?”, i.e. which combination of ES objectives, contextual preconditions, and design elements has led to successful outcomes? As explained above, in answering this question we are handicapped by an extreme scarcity of rigorous PES impact evaluations, and by a sample size that moderately limits our degrees of freedom in statistical analysis. In response, we attempt in this section to answer econometrically a more limited sub-question: What key PES design factors may predict whether a PES scheme achieves at least some environmental additionality? An emerging literature on the principles of environmentally effective PES design points to particularly three critical factors of high potential [44–48]:

1. *Spatial targeting of contracts*—vis-à-vis hot-spots of high ES intensity, and high threat (leverage of change), respectively: by using pre-identified spatial filters to give explicitly higher focus to areas of potentially high ES gains (e.g. biodiversity hotspots) and high leverage (e.g. current deforestation hotspots), the chances for making a measurable environmental difference increase;
2. *Differentiated payments—*vis-à-vis variable provision costs across ES providers: whenever ES providers are heterogeneous in their profit opportunities (due to different asset holdings and technologies, market access, preferences, etc.), then offering them different payments levels (or even variable contract types) leads to greater cost efficiency than paying everybody the same (per hectare, family), thus potentially stretching the scheme’s environmental gains;
3. *Conditionality degree*—the implementer’s combined efforts to monitor and sanction incompliance: PES schemes that are perceived by ES providers to be ill-monitored and –enforced will often eventually lead to widespread non-compliance, as cashing in on PES while following ‘business as usual’ becomes a profitable cheating strategy. The hypothesis here is that PES schemes which go serious about implementing this quintessential PES feature will also tend to perform better with respect to their environmental outcomes.

For spatial targeting (i), we distinguished three progressive levels: a) no targeting, b) either ES density or threat targeted; c) both density and threat targeted. Differentiated payments (ii) were classified binarily, according to whether or not more than one single payment level (predominantly per-hectare, but also per-household payments) was used. Conditionality (iii) we constructed as the product of indices for documented monitoring and sanctioning efforts, respectively.

In addition, we included three control variables in the estimation. First, the public vs. private commercial and non-commercial funding distinction proved its importance in the preceding analysis. Second, baselines and additionality assessments tend to differ substantially whether PES are “asset-building” (i.e. paying for added environmental value, such as tree planting) or “activity-restricting”, (i.e. made for avoiding projected damage, such as reducing deforestation) [40]. Finally, the time elapsed since project start could also be stage-setting, e.g. in terms of learning-by-doing effects on environmental effectiveness being allowed to kick in as the implementation process proceeds.

Table 3 shows the result of the binary logistical regression testing the predictive level of the above described critical design variables on environmental additionality for the 51 cases for which it was possible to obtain an estimation of additionality. Overall, the model has a robust fit (Cox & Snell R^2^ = 0.52; Nagelkerke R^2^ = 0.79) correctly predicting 97% of additionality and 83% of no additionality cases (overall predictive accuracy of 94%); the Hosmer-Lemeshow test to assess the null hypothesis that the model prediction does not fit perfectly with the observed data was not significant (H-L p = 0.55), confirming the goodness of fit of the model. Multicollinearity checks are also robust indicating no collinearity among the variables included in the model (Tolerance>0.2; VIF<3; S7 and S8 Tables; VIF: Variance Inflation Factor).

**Table 3:** Binary logistic regression model predicting the degree of PES environmental additionality.

	Model I		Model II	
*Variable*	*Coefficient*	*Standard error*	*Coefficient*	*Standard error*
Activity paid	5.25**	2.41	5.83*	3.45
(Dummy: 0 = Conservation; 1 = Asset building)				
Payments diversification (level = 0)	5.11**	2.50	4.64*	2.83
(Dummy: 0 = No; 1 = Yes)				
Spatial targeting	6.74*	3.58	9.31*	5.58
(Ordinal: 2 = Threat and ES density)				
Conditionality	1.56**	0.80	1.45*	0.85
(Ordinal: Monitoring*Sanction)				
Sector financed				
(Nominal: 1 = Private profit; 2 = Private non-profit)				
Level = Private profit	6.92	22.30	9.29	72.33
Level = Private non-profit	-3.82	3.25	-6.99	5.50
Time (years since PES scheme)	-0.40**	0.18	-0.53*	0.30
Additionality assessment precision			1.30	1.04
(Ordinal: 1 = very weak; 5 = very strong)				
Constant	-10.54*	5.82	-13.28*	7.41
N = 51				
-2 Log likelihood =	18.00		14.78	
Cox & Snell R^2^	0.52		0.55	
Nagelkerke R^2^	0.79		0.83	
Correct predictions	94.1%		96.1	
H-L test p	0.55		1	

*/**Statistical significance at 10% and 5%.

Starting with the control variables (a) in Model I, we see that it makes the expected difference for assessed additionality whether the PES scheme is asset-building versus preventively conserving nature by avoiding projected pressures. This expresses probably that it is much easier to verify the achievement of an additional outcome when an asset has been added (e.g. planting a tree) than when a projected damage has been avoided (e.g. leaving a threatened tree standing) (dummy significant at 5% level). Interestingly, the two sectoral control variables do not come out as significant, in spite of having been flagged as important determinants of clusters in the above. We attribute this to the inclusion of design variables: much of what we see as sector differences may hide different PES design principles. Finally, the implementation time variable is significant (5% level), but with an unexpected negative sign, indicating that older schemes tend to be less additional than more recent ones. Since most of the schemes are still ongoing, implementation time thus widely comes to reflect the calendar year when the scheme was implemented. While there is thus little evidence of learning-by-doing impacts *within* each PES scheme, implementers may indeed learn more from each other (e.g. through various PES networks), so that newer PES schemes are able to avoid some features that have not worked well for other implementers in the past, in terms of reaching their environmental objectives.

Turning now to the three PES design variables, they were all estimated with the expected positive sign (i.e. increasing additionality), though with slightly different levels of significance: payment diversification and the degree of conditionality had a strong effect (significant at 5% level), while spatial targeting narrowly misses that threshold (significant at 10% level). This is an important finding, which reconfirms the recommendations for implementation that are being put forward in the more theoretically orientated PES literature.

Our results could potentially have been affected by the precision level of the environmental additionality estimations. To control for the sensitivity of the model to this factor, we run the previous model including the created ordinal variable describing the precision of additionality measures (Model II in Table 3). The resulting model is robust (Cox & Snell R^2^ = 0.55; Nagelkerke R^2^ = 0.83; overall predictive accuracy of 96%; no multicollinearity) and confirms the predictive significance of the three critical design factors: Diversification of payments, spatial targeting and conditionality.

## Discussion and Conclusion

Meta-analyses can help us recognizing global empirical patterns of PES. As the PES literature flourishes and new case studies are continuously being added, we have the opportunity to gain more knowledge about commonalities in implementation and outcomes. In this article, we took advantage of the expansion of PES case studies in recent years to construct a global database. We used a fairly narrow and explicit definition of PES, in order to identify comparable schemes. The cases selected show a balanced number of schemes by geographical region, ecosystem type, ecosystem service and economic sector involved, showing that the systematic review procedure succeeded in limiting the risk of selection bias. For instance, the limited number of African PES schemes included in our study reflects the actual slower up-take of PES in this continent. We included quantitative and qualitative data from what we see functionally as “genuine” PES schemes, using a narrow PES definition that ensures comparability. We also filtered out cases without the availability of sufficient reliable documentation. We then conducted some exploratory analysis of the emerging patterns of implementation, going from purely descriptive bivariate statistics to principal component analysis of emerging variable clusters, and finally to test some theoretically sustained hypotheses regarding the association of alleged key PES design variables with positive environmental additionality.

From our descriptive analysis, we noted significant differences between publicly and privately financed PES schemes (e.g. public schemes feature larger in area and more costly schemes), but also between the different ES that these schemes target. Public sector PES participation is high in Europe and Asia (with a tradition for public-sector environmental management), yet very low in Sub-Saharan Africa, where public sector institutions have lower capacity to organize PES schemes. Latin America, the prime region of PES implementation, displays a large variety of arrangements.

Next, we identified through categorical principal component analysis three dominating clusters, which we label “agri-environmental public PES”, “NGO-led biodiversity PES”, and “private commercial carbon PES”. Each of these interacts with a set of variables (ecosystem, target ES, lead actors, etc.). While we thus reconfirm the hypothesis from the pre-existing PES literature that public and private PES differ significantly, our results also point to important differences between the two types of private PES schemes: the typical commercial PES (private for-profit company) and the non-commercial (non-governmental not-for-profit organizations) exhibit distinct patterns. Still, also some permeability between the three groups remains, showing that PES implementation is also the result of a hybridization and cooperation between public and private sectors.

Finally, we also attempted to scrutinize which PES design factors influenced environmental outcomes, as measured by a binarily defined proxy of environmental additionality. We confirmed the significance of spatial targeting (for ES density and threat) and, to a statistically somewhat lesser extent, of payment differentiation (as opposed to uniform payments) and the degree of conditionality (monitoring and sanctioning efforts applied). Payments for building environmental assets were also more likely to be additional than payments for avoiding damages. Interestingly, the public vs. private sector distinction, applied here as a control variable, no longer came out as significant. This could imply that the main differences in outcomes between public and private PES manifest themselves through divergences in technical PES design principles, rather than being *sui generis* differences. Our additionality findings are robust to the sensitivity analysis with respect to the precision in measuring environmental additionality: stronger precision does not condition the validity of the model, and the key design variables remain significant.

As so often in complex interactions between social and biophysical systems, could there potentially be problems of endogeneity in the relations we tested for in our binary logistical regression analysis? For instance, additionality targets may certainly from the outset be lower in public PES schemes, which tend to have more side-objectives. However, this should be controlled for by our sector variables. In principle, we believe that environmental additionality as a response variable constitutes a true ‘bottom line’ of the interplay between PES contexts and the design of PES interventions. We are thus confident that endogeneity does not constitute a major problem, if any. Yet, as a precautionary measure and sensitivity check, we also ran the model at three different probability cut-off levels (0.25, 0.50 and 0.75), in order to capture the influence of the *ex-ante* probability of the environmental additionality in the model. We find no changes in model descriptors although the highest accuracy is obtained with the equal-probability assumption of 0.50 (S9 Table). This reinforces our conviction that our current model specification is robust.

For future research, we believe our global-comparative analysis could eventually be improved by including more cases, as they become available, and by more consolidated information about rigorously evaluated key PES outcomes, in both environmental and socioeconomic terms. However, the current scarcity of rigorous impact evaluations applies not only to PES, but basically to any conservation tool other than protected areas [32,41]. While our additionality measure and some of the design proxies are admittedly still rough approximations, our results can be seen as a first set of pointers, to be tested subsequently in more sophisticated ways with more and better data.

Nevertheless, our analyses give an interesting indication that, after controlling for various contextual factors, the application of the best PES design principles may add value to the environmental outcomes. This represents a call for greater efforts of using state-of-the-art principles to make PES design more sophisticated, e.g. in terms of monitoring, targeting and differentiation. As a recommendation, this will probably not be favored across the board by all PES implementers. Yet, to put it conversely, our results indicate that there may be ample efficiency costs attached to the over-simplification of policies and interventions in environmental incentive programs such as PES.

## Supporting Information

S1 Table References used for the description of PES selected schemes. (DOCX)

S2 Table Fitness to a canonical PES scheme composite indicator. (DOCX)

S3 Table Additionality assessment evidence. (DOCX)

S4 Table Additionality assessment summary. (DOCX)

S5 Table ANOVA and t-test means differences for payments amounts and size of PES programs, by sector and targeted ES. (DOCX)

S6 Table Rotated component loadings from a two-dimensional CatPCA on 26 PES variables, with all variables analysed nominally excepted market composition and FitCanPES level. (DOCX)

S7 Table Pearson correlation levels and significance between the predictor variables of the bivariate additionality model. (DOCX)

S8 Table Collinearity analysis for the predictor variables of the bivariate additionality model. (DOCX)

S9 Table Logistic regression models with cut-off level set at 0.25, 0.50 and 0.75. (DOCX)

S1 Database PES schemes and descriptors. (XLSX)
