# Supplementary material for: Global Patterns in the Implementation of Payments for Environmental Services
Source: PLoS One. 2016 Mar 3;11(3):e0149847. doi: 10.1371/journal.pone.0149847 (PMC4777491; doi:10.1371/journal.pone.0149847)
Supplement: S1 Table — (DOCX) [file pone.0149847.s001.docx]

**S1 Table. References used for the description of PES selected schemes.**

| Id_PES | Id_Label | PES_description | Supporting references |
| --- | --- | --- | --- |
| 1 | NegB | Los Negros, Bolivia | Asquith NM, Vargas MT, Wunder S. (2008). Selling two environmental services: In-kind payments for bird habitat and watershed protection in Los Negros, Bolivia. Ecological Economics 65:675–684.  Robertson, N., and S. Wunder. (2005). Fresh tracks in the forest: Assessing incipient payments for environmental services initiatives in Bolivia. Bogor: CIFOR. |
| 2 | PimE | Pimampiro, Ecuador | Wunder S, Albán M. (2008). Decentralized payments for environmental services:  The cases of Pimampiro and PROFAFOR in Ecuador. Ecological Economics 65: 685-698.  Cordero-Camacho D. (2008). Esquemas de pagos por servicios ambientales para la conservación de cuencas hidrográficas en el Ecuador. Investigación Agraria: Sistemas y Recursos Forestales 17(1) 54-66. |
| 3 | ProE | PROFAFOR, Ecuador | Wunder S, Albán M. (2008). Decentralized payments for environmental services:  The cases of Pimampiro and PROFAFOR in Ecuador. Ecological Economics 65: 685-698.  Albán M, Argüello M. (2004). Un análisis de los impactos sociales y económicos de los proyectos de fijación de carbonoen el Ecuador: el caso de Profafor-Face. Mercados para servicios ambientales. International Institute for Environment and Development, London, UK. |
| 4 | VitF | Vittel (Nestlé Waters), France | Perrot-Maître D. (2006). The Vittel payments for ecosystem services: a “perfect” PES case? International Institute for Environment and Development, London, UK.  Perrot-Maître D. (2005). Investing in protection of ecosystem services: a business opportunity for Vittel (Nestlé Waters), France. Paper presented at the ZEF–CIFOR workshop on payments for environmental services in developed and developing countries, Titisee, Germany, 15–18 June 2005. |
| 5 | SloCh | Sloping Land Conversion, China | Bennett MT. (2008). China's sloping land conversion program: Institutional innovation or business as usual? Ecological Economics 65:699-711.  Xu J, Tao R, Xu Z, Bennett MT. (2010). China’s Sloping Land Conversion Program: Does Expansion Equal Success? Land Economics 86(2):219–244. |
| 6 | HydroMX | Payments for Hydrological Environmental Services (PSAH), Mexico | Muñoz-Piña C, Guevara A, Torres JM, Braña J. (2008). Paying for the hydrological services of Mexico's forests: Analysis, negotiations and results. Ecological Economics 65:725-736.  Alix-Garcia JM, Shapiro EN, Sims K. (2012). Forest Conservation and Slippage: Evidence from Mexico’s National Payments for Ecosystem Services Program. Land Economics 88 (4): 613–638. |
| 7 | ConsUS | Conservation Reserve Program (CRP), USA | Claassen R, Cattaneo A, Johansson R. (2008). Cost-effective design of agri-environmental payment programs: U.S. experience in theory and practice. Ecological Economics 65:737-752.  Leathers N, Harrington L. (2000). Effectiveness of conservation reserve programs and land ‘slippage’ in southwestern Kansas. Professional Geographer 52:83–93. |
| 8 | EnvUS | Environmental Quality Incentives Program (EQIP), USA | Claassen R, Cattaneo A, Johansson R. (2008). Cost-effective design of agri-environmental payment programs: U.S. experience in theory and practice. Ecological Economics 65:737-752.  Cattaneo A. (2003). The pursuit of efficiency and its unintended consequences: contract, withdrawals in the environmental quality incentives program. Review of Agricultural Economics 25:449–469.  U.S. Department of Agriculture, Economic Research Service (USDAERS). (2014). Environmental Quality Incentives Program Data. Online document http://www.ers.usda.gov/Data/eqip last accessed February 19, 2014. |
| 9 | SenUK | Environmentally Sensitive Area (ESA) , United Kingdom | Dobbs TL, Pretty, J. (2008). Case study of agri-environmental payments: The United Kingdom. Ecological Economics 65:765-775.  Crabtree B, Thorburn A, Chalmers N, Roberts D, Wynn G, Barron N, Macmillan D, Barraclough F. (2000). Socio-economic and Agricultural Impacts of the Environmentally Sensitive Areas (ESA) Scheme in Scotland. A Report for the Scottish Executive Rural Affairs Department. Macaulay Land Use Research Institute, with Bell-Ingram Rural and University of Aberdeen, Craigiebuckler, Aberdeen. |
| 10 | CouUK | Countryside Stewardship Scheme (CSS), United Kingdom | Dobbs TL, Pretty, J. (2008). Case study of agri-environmental payments: The United Kingdom. Ecological Economics 65:765-775.  Crabb J, Short C, Temple M, Winter M, Augustin B, Dauven A (2000) Economic Evaluation of the Countryside Stewardship Scheme. Prepared for Ministry of Agriculture, Fisheries and Food (MAFF), Economics (Resource Use) Division. Cheltenham and Glouchester College of Higher Education and ADAS. |
| 11 | NotrG | Northeim model project, Germany | Klimeka S, Richter gen. Kemmermanna A, Steinmann HH, Freese J, Isselstein J. (2008). Rewarding farmers for delivering vascular plant diversity in managed grasslands: A transdisciplinary case-study approach. Biological Conservation 141:2888–2897.  Grolleau G, McCann LMJ. (2012). Designing watershed programs to pay farmers for water quality services: Case studies of Munich and New York City. Ecological Economics 76 :87–94. |
| 12 | CamZ | CAMPFIRE, Zimbabwe | Frost PGH, Bond I. (2008). The CAMPFIRE programme in Zimbabwe: Payments for wildlife services. Ecological Economics 65:776-787.  Dunham KM, Davies C,Muhwandagara K. (2003). Area and Quality of Wildlife Habitat in Selected CAMPFIRE Districts. WWF-SARPO and the CAMPFIRE Association, Harare, Zimbabwe, (mimeo). |
| 13 | LavCo | CIPAV-Río La Vieja, Colombia | Zapata A, Murgueitio E, Mejía C, Zuluaga AF, Ibrahim M. (2007). Efecto del pago por servicios ambientales en la adopción de sistemas silvopastoriles en paisajes ganaderos de la cuenca media del río La Vieja, Colombia. Agroforesteria en las Américas 45:86-92.  Pagiola S, Rios AR, Arcenas A. (2010). Poor household participation in payments for environmental services: Lessons from the Silvopastoral Project in Quindío, Colombia. Environmental and Resource Economics 47:371-394. |
| 14 | ChaE | Chachis, Ecuador | Wendland K, Suárez L. (2009). Incentivos a cambio de servicios ambientales y colectividad de la tenencia de la tierra: Lecciones de Ecuador e Indonesia. USAID Land Tenure Center, Tenure Brief no. 9.  Speiser S, Bauer K, Villacreset D. (2009). Buenas Prácticas Conservación y Desarrollo: una experiencia de los Chachi en el Noroccidente Ecuatoriano. GTZ. Available at <http://www.unl.edu.ec/agropecuaria/wp-content/uploads/2012/03/sp-buenas_practicas-areas-protegidas-Chachi-ecuador1.pdf>. |
| 15 | ChaCo | Chaina, Colombia | Borda-Almanza CA, Moreno RP, Wunder S. (2009). Pagos por Servicios Ambientales en Marcha: La Experiencia en la Microcuenca de Chaina, Departamento de Boyacá, Colombia. CIFOR unpublished report 54p.  Moreno-Sanchez, Rocio, Jorge Higinio Maldonado, Sven Wunder, and Carlos Borda-Almanza. (2012). Heterogeneous users and willingness to pay in an ongoing payment for watershed protection initiative in the Colombian Andes. Ecological Economics 75:126-134. |
| 16 | ProcCo | Procuenca, Colombia | Cardona-Calle DA. (2008). Implementación de mecanismos de compensación por servicios ambientales: incentivos y captura de carbono: Estudio de caso Procuenca, Colombia. Available at <http://globalforestcoalition.org/wp-content/uploads/2010/11/Estudiodecaso-Procuenca-Colombia1.pdf>.  Erazo J, Benjumea F. (2004). Análisis de la aplicación de la exoneración del impuesto predial como incentivo para la conservación en Manizales. Instituto Von Humbodt, Bogota, Colombia. 38p. |
| 17 | CelE | Celica, Ecuador | Yaguache-Ordóñez R. (2013). Los acuerdos voluntarios para la conservación y restauración de bosques. Presentation at Sustainable Forest Production international congress, Quito, Ecuador, 12–13 June 2013.  Raes, Leander, Eduardo Rengel, and José Romero. (2012). Inter-municipal cooperation in watershed conservation through the establishment of a regional water fund–FORAGUA–in Southern Ecuador. |
| 18 | ChacE | Chaco, Ecuador | Cordero-Camacho D. (2008). Esquemas de pagos por servicios ambientales para la conservación de cuencas hidrográficas en el Ecuador. Investigación Agraria: Sistemas y Recursos Forestales 17(1) 54-66. |
| 19 | RichS | Richtersveld, Sudafrica | Robinson R. (1998). Community partnership in the Richtersveld National Park, South Africa. Paper presented in the Scandinavian Seminar College Workshop in Abidjan, Ivory Coast, November 9 - 11, 1998. |
| 20 | KitK | Kitengela, Kenya | Yatich T, Said M, Swallow B, Sononka J. (2008). Kitengela Wildlife Lease Programme: Is it Realistic, conditional, pro-poor and voluntary? East and Southern Africa Katoomba Group Regional Workshop on Taking stock and char  ting a way forward: Payments for Ecosystem Services in Africa, 16-17 September 2008, Dar-es-salaam and Morogoro, Tanzania.  Osano P, de Leeuw, J, Said M. (2012). Wildlife PES Schemes and Pastoral livelihoods in Arid & Semi-Arid Lands (ASALs) in Kenya. Presentation at the workshop Restoring Value to Grasslands, 7-10 may 2012, Brasilia, Brazil. |
| 21 | MenM | Menabe, Madagascar | Sommerville M, Jones JGP, Rahajaharison M, Milner-Gulland EJ. (2010). The role of fairness and benefit distribution in community-based Payment for Environmental Services interventions: A case study from Menabe, Madagascar. Ecological Economics 69: 1262–1271. |
| 22 | TurtT | Sea turtle nest, Tanzania | Ferraro PJ. (2007). Performance Payments for Sea Turtle Nest Protection in Low income Nations: a case study from Tanzania. Southwest Fisheries Science Center,  National Marine Fisheries Service National Oceanic and Atmospheric Administration. Available at <http://www2.gsu.edu/~wwwcec/docs/doc%20updates/NOAA%20Paper%20TZ%20Final%20Draft%20June%202007.pdf>.  Ferraro PJ, Gjertsen H. (2009). A Global Review of Incentive Payments for Sea Turtle Conservation. Chelonian Conservation and Biology 8(1):48-56. |
| 23 | BirwCa | Bird watch & ecotourism, Cambodia | Clements T, Ashish J, Nielsen K, An D, Tan S, Milner-Gulland EJ. (2010). Payments for biodiversity conservation in the context of weak institutions: Comparison of three programs from Cambodia. Ecological Economics 69: 1283–1291.  Clements T, John A, Nielsen K, Vicheka C, Sokha E, Piseth M. (2008). Tmatboey Community-based Ecotourism Project, Cambodia. USAID, TransLinks. 56p. |
| 24 | BirnCa | Bird nest protection, Cambodia | Clements T, Ashish J, Nielsen K, An D, Tan S, Milner-Gulland EJ. (2010). Payments for biodiversity conservation in the context of weak institutions: Comparison of three programs from Cambodia. Ecological Economics 69: 1283–1291.  Clements T, Rainey H, An D, Rours V, Tan S, Thong S, Sutherland WJ, Milner-Gulland EJ. (2013). An evaluation of the effectiveness of a direct payment for biodiversity conservation: The Bird Nest Protection Program in the Northern Plains of Cambodia. Biological Conservation 157:50–59. |
| 25 | SocE | SocioBosque, Ecuador | de Koning F, Aguiñaga M, Bravo M, Chiu M, Lascano M, Lozada T, Suarez L. (2011) Bridging the gap between forest conservation and poverty alleviation: the Ecuadorian Socio Bosque program. Environmental Science & Policy 14:531–542.  Krause, Torsten, Wain Collen, and Kimberly A Nicholas. (2013). Evaluating Safeguards in a Conservation Incentive Program: Participation, Consent, and Benefit Sharing in Indigenous Communities of the Ecuadorian Amazon. Ecology and Society 18 (4):1. |
| 26 | JesH | Jesús de Otoro, Honduras | Kosoy N, Martinez-Tuna M, Muradian R, Martinez-Alier J. (2007). Payments for environmental services in watersheds: Insights from a comparative study of three cases in Central America . Ecol Econ 61 :446-455.  Ardón-Mejía M, Martínez MA. (2006). Evolución de las Experiencias de Servicios Ambientales Hídricos en Honduras. Los casos de los municipios de Jesús de Otoro (Intibucá) y Campamento (Olancho). Programa para la Agricultura Sostenible en Laderas de América Central PASOLAC. Technical series no. 495. |
| 27 | HerCR | Heredia, Costa Rica | Kosoy N, Martinez-Tuna M, Muradian R, Martinez-Alier J. (2007). Payments for environmental services in watersheds: Insights from a comparative study of three cases in Central America . Ecol Econ 61 :446-455.  Barrantes G, Gamez L. (2007). Programa de Pago por Servicio Ambiental Hídrico de la Empresa de Servicios Públicos de Heredia. World Bank unpublished report, Washington D.C., USA. 20p. |
| 28 | SanNC | San Pedro del Norte, Nicaragua | Kosoy N, Martinez-Tuna M, Muradian R, Martinez-Alier J. (2007). Payments for environmental services in watersheds: Insights from a comparative study of three cases in Central America . Ecol Econ 61 :446-455.  Pagiola S. (2008). Can the poor participate in payments for environmental services? Lessons from the Silvopastoral Project in Nicaragua. Environment and Development Economics 13:299–325. |
| 29 | KulN | Kulekhani, Nepal | Khatri DB. (2009). Compromising the environment in Payments for Environmental Services? An institutional analysis of mechanisms for sharing hydroelectricity revenue in Kulekhani watershed, Nepal. Master thesis, Insternational Institute of Social Studies, The Hague, Netherlands. 51p.  Joshi, Laxman. (2011). A community-based PES scheme for forest preservation and sediment control in Kulekhani, Nepal. In FAO “Payments for Ecosystem Services and Food Security”, 198-203. FAO, Rome, Italy. |
| 30 | DonV | Da Nhim PWS, Dong Nai watershed, Vietnam | Wertz-Kanounnikoff S, Rankine H. (2008). How can governments promote strategic approaches to payments for environmental services (PES)? An exploratory analysis for the case of Vietnam. Natural Resources no. 3, IDDRI, Paris, France.  Bich-Thuy NT, Thanh NC, Phuong-Thao N, Nam PT, Bonnardeaux D, Cottrill-Riedel D. (2011). Lam Dong Province PES Technical Working Group Payment for Forest Environmental Services: A Case Study on Pilot Implementation in Lam Dong Province, Vietnam 2006-2010. Winrock International. Available at <http://jeevika.org/bamboo/1k-payment-for-forest-environmental-services.pdf>. |
| 31 | SonV | Son La PWS, Vietnam | Bich-Thuy NT, Thanh NC, Phuong-Thao N, Nam PT, Bonnardeaux D, Cottrill-Riedel D. (2011). Lam Dong Province PES Technical Working Group Payment for Forest Environmental Services: A Case Study on Pilot Implementation in Lam Dong Province, Vietnam 2006-2010. Winrock International. Available at <http://jeevika.org/bamboo/1k-payment-for-forest-environmental-services.pdf>.  Minh Ha H, Van-Noordwijk M , Thu-Thuy P. (2008). Payment for environmental services: Experiences and lessons  in Vietnam. Hanoi, Vietnam. World Agroforestry Centre (ICRAF). 33 p. |
| 32 | OacI | Oach Kalan - Kuhan mini micro watershed, India | Agarwal, C, Tiwari, S, Borgoyary, M, Acharya, A, Morrison, E. (2007). Fair deals for watershed services in India. Natural Resource Issues no. 10. International Institute for Environment and Development. London, UK. |
| 33 | ZapMX | Saltillo, Zapaliname, Mexico | Laurans Y, Leménager T, Aoubid S. (2011). Agence Française pour le Développement. Paris, France. |
| 34 | SimT | Simanjiro valley, Tanzania | Nelson F. (2008). Developing Alternative Frameworks for Community-based Conservation: Piloting Payments for Environmental Services (PES) in Tanzania’s Simanjiro Plains. USAID TransLinks. 38p.  Laurans Y, Leménager T, Aoubid S. (2011). Agence Française pour le Développement. Paris, France. |
| 35 | BioN | NRCB management, Namibia | Weaver C, Petersen T. (2008). Namibia communal area conservancies. Best Practices in Sustainable Hunting pp. 48–52.  Weaver LC, Petersen T, Diggle R, Matongo G. (2009). Achievements and Practical Lessons Learned From A Decade of Wildlife Utilization In Namibia’s Communal Area Conservancies. WWF. |
| 36 | CatUS | Catskills, NYC, USA | Grolleau G, McCann LMJ. (2012). Designing watershed programs to pay farmers for water quality services: Case studies of Munich and New York City. Ecological Economics 76:87–94.  Laurans Y, Leménager T, Aoubid S. (2011). Agence Française pour le Développement. Paris, France. |
| 37 | UlgT | Tanzania, PWS | Lopa D, Mwanyoka I, Jambiya G, Massoud T, Harrison P, Ellis-Jones M, Blomley T, Leimona B, van-Noordwijk M,  Burgess ND. (2012). Towards operational payments for water ecosystem services in Tanzania: a case study from the Uluguru Mountains. Oryx 46(1):34-44.  Branca, Giacomo, Leslie Lipper, Bernardete Neves, Dosteus Lopa, and Iddi Mwanyoka. (2011). Payments for watershed services supporting sustainable agricultural development in Tanzania. The Journal of Environment & Development 20 (3):278-302. |
| 38 | SilNC | Silvopastoril, Nicaragua | Marín Y, Ibrahim M, Villanueva C, Ramírez E, Sepulveda C. (2006). Los impactos de un proyecto silvopastoril en el cambio de uso de la tierra y alivio de la pobreza en el paisaje ganadero de Matiguás, Nicaragua. Agroforesteria en las Américas no. 45:109-116.  Ibrahim M, Villanueva CP, Casasola F. (2007). Sistemas silvopastoriles como una herramienta para el mejoramiento de la productividad y rehabilitación ecológica de paisajes ganaderos en centro américa. Arch. Latinoam. Prod. Anim. Vol. 15:73-87. |
| 39 | SilCR | Silvopastroril, Costa Rica | Pagiola S. (2010). Desafíos y Oportunidades para el desarrollo de Pagos por Servicios Ambientales en el Sector Ganadero. VI Agroforestry Congress on Cattle Sustainable Production, Ciudad de Panama, Panamá, 28-30 September, 2010.  Ibrahim M, Gobbi J, Casasola F, Chacón M, Ríos N, Tobar D, Villanueva C, Sepúlveda C. (2007). Enfoques alternativos de pagos por servicios ambientales: Experiencia del proyecto Silvopastoril. World Bank, Washington D.C., USA. |
| 40 | CidIS | Cidanau watershed PES scheme, Indonesia | Leimona B, Pasha R, Rahadian NP. (2010). The livelihood impacts of incentive payments for watershed management in Cidanau watershed, West Java, Indonesia. Payments for Environmental Services, Forest Conservation and Climate Change Livelihoods in the REDD? Eds. Tacconi L, Mahanty S and Suich H.  Suyanto S, Leimona B, Permana RP, Chandler FJC. (2005). Review of the development environmental services market in Indonesia. World Agroforestry Center. Available at <http://www.worldagroforestrycentre.org/sea>. |
| 41 | TreU | Uganda, Trees for Global Benefits Programme | German LA, Ruhweza A, Mwesigwa R, Kalanzi C. (2010). Social and environmental footprints of carbon payments: a case study from Uganda. Payments for Environmental Services, Forest Conservation and Climate Change Livelihoods in the REDD? Eds. Tacconi L, Mahanty S and Suich H.  BioClimate Research and Development and EcoTrust. (2006). Trees for Global Benefit: A Plan Vivo Project. Bushenyi Field Trip Report, Uganda, 17 to 20 July, 2006. Edinburgh and Kampala: BR&D and EcoTrust. |
| 42 | FirPH | Philippines, No Fire Bonus Scheme | Soriaga R, Annawi D. (2010). The ‘No-Fire Bonus’ Scheme in Mountain Province, Cordillera Administrative Region, Philippines. Payments for Environmental Services, Forest Conservation and Climate Change Livelihoods in the REDD? Eds. Tacconi L, Mahanty S and Suich H.**rt que te mande......de** |
| 43 | TreMZ | Mozambique, carbono. | Hegde R. (2010). Payments for ecosystem services and farm household behaviour: The case of carbon in Mozambique’s  Agroforests. PhD University of British Columbia.  Jindal R. (2012). Reducing Poverty Through Carbon Forestry? Impacts of the N’hambita Community Carbon Project in Mozambique. World Development 40(10):2123–2135. |
| 44 | TreMX | Scolel Té | Quechulpa-Montalvo S, Esquivel-Bazán E, Fournier S. (2011). Scolel’ Te Program Plan Vivo Annual Report 2010. Available at <http://www.planvivo.org/>.  AMBIO. (2012). The Scolel’Te Programme 2011 Annual Report Reporting period: March 2011 to March 2012. Available at <http://ambio.org.mx/>. |
| 45 | MonMX | Monarch | Honey-Rosés J, Baylis K, Ramirez I. (2011). A Spatially Explicit Estimate of Avoided Forest Loss. Conservation Biology 25(5):1032–1043.  Honey-Rosés J, Lopez-Garcia J, Rendon-Salinas E, Peralta-Higuera A, Galindo-Leal C. (2009). To pay or not to pay? Monitoring performance and enforcing conditionality when paying for forest conservation in Mexico. Environmental Conservation 36 (2): 120–128. |
| 46 | PwsG | PWS en Munich | Grolleau G, McCann LMJ. (2012) Designing watershed programs to pay farmers for water quality services: Case studies of Munich and New York City. Ecological Economics 76:87–94.  Simonet N. (2005). L'agriculture biologique au service de l'eau. Report. |
| 47 | SumIn | Sumberjaya AF conservation auction | Leimona B, Kelsey-Jack B, Lusiana B, Pasha R. (2009) Designing A Procurement Auction For Reducing Sedimentation: A Field Experiment In Indonesia. Economy and Environment Program for Southeast Asia research report no. 10.  Suyanto, S. (2007). Lessons on the conditional tenure and RiverCare schemes in Sumberjaya, Indonesia: conditionality in payment for environmental services. Insight: Notes from the Field. Bangkok, Tailandia. RECOFTC, Centro Mundial de Agrosilvicultura (ICRAF) y Winrock International India (WII):29-35. |
| 48 | RefK | Reforestation conservation auction in Kenya | Wünscher T, Khalumba M, Holm-Müller K, Büdenbender M. (2012) The Cost-Effectiveness of Combining Reforestation Auctions with Performance Based Payments – A Field Trial in Rural Kenya. Paper presented at the congress of the International Society of Ecological Economics, Rio de Janeiro, 16-19 June 2012.  Khalumba M, Wünscher T, Wunder S, Büdenbender M, Holm-Müller K. (2014). Combining Auctions and Performance-Based Payments in a Forest Enrichment Field Trial in Western Kenya, Conservation Biology 28(3): 861–866. |
| 49 | FuqCo | Fuquene, Colombia | Quintero M, Otero W. (2006) Mecanismo de financiación para promover Agricultura de Conservación con pequeños productores de la cuenca de la laguna de Fúquene. Su diseño, aplicación y beneficios. Centro Internacional de la Papa, Lima, Peru.  Garzón, A. (2009). Estado de la acción sobre los mecanismos de financiamento de la protección o recuperación de servicios ambientales hidrológicos generados en los Andes. Quito: Ecodesión. |
| 50 | KmpfB | Noel Kempff Mercado REDD+ project | Asquith NM, Vargas-Ríos MT, Smith J. (2002) Can forest-protection carbon projects improve rural livelihoods? Analysis of the Noel Kempff Mercado climate action project, Bolivia. Mitigation and Adaptation Strategies for Global Change 7: 323–337.  Pereira, S. (2010). Payment for Ecosystem Services in the Amazon Forest: How Can Conservation and Development be Reconciled? The Journal of Environment & Development. 19(2): 171-190. |
| 51 | PesCR | Payments for Environmental Services (PSA), Costa Rica | Pagiola S. (2008) Payments for environmental services in Costa Rica. Ecological Economics 65 :712-724.  Arriagada RA, Ferraro PJ, Sills EO, Pattanayak SK, Cordero-Sancho S. (2012) Do Payments for Environmental Services Affect Forest Cover? A Farm-Level Evaluation from Costa Rica. Land Economics (88)2:382-399. |
| 52 | MunCo | CIPAV- La Salvajina & PNN Munchique, Colombia | CIPAV (2007) Desarrollo del pago por servicios ambientales para la conservacion y restauración de ecosistemas en el corredor biológico y multicultural Munchique Pinche. CIPAV, Cali, Colombia. |
| 53 | MakMa | Makira WCS Madagascar | Brimont, L., Ezzine-de-Blas, D., Karsenty, A., Tambaza, F., Toulon, A., Rasolofonirina, G., Razanamihanta, E. (2015). Achieving REDD+ objectives along with equity principles in Madagascar: a critical analysis of Makira project’s development strategy. Forests 2015 6: 748-768. |
| 54 | LrcB | Landrace conservation payments Bolivia & Peru | Narloch U, Pascual U, Drucker AG (2011) Cost-effectiveness targeting under multiple conservation goals and equity  considerations in the Andes. Environmental conservation. Environmental Conservation 38(4):417-425.  Narloch, U. (2011). Payments for agrobiodiversity conservation services: how to make incentive mechanisms work for conservation PhD, Corpus Christi College, University of Cambridge, Cambridge. |
| 55 | BfeB | Bolsa Floresta - Brasil | Börner, J. , S. Wunder, F Reimer, R.K. Bakkegaard, V. Viana, J Tezza, T. Pinto, L. Lima, and S. Marostica. (2013). Promoting Forest Stewardship in the Bolsa Floresta Programme: Local Livelihood Strategies and Preliminary Impacts. Rio de Janeiro, Manaus & Bonn: Center for International Forestry Research (CIFOR), Fundação Amazonas Sustentável(FAS). Zentrum für Entwicklungsforschung (ZEF), University of Bonn. |
