# Supplementary material for: Global Patterns in the Implementation of Payments for Environmental Services
Source: PLoS One. 2016 Mar 3;11(3):e0149847. doi: 10.1371/journal.pone.0149847 (PMC4777491; doi:10.1371/journal.pone.0149847)
Supplement: S2 Table — (DOCX) [file pone.0149847.s002.docx]

**S2 Table. Fitness to a canonical PES scheme composite indicator.**

Variables and scores defining the composite indicator ‘Fitness to a canonical PES definition’.

| Criteria | Values | Values definition | | | | Reference | | |
| --- | --- | --- | --- | --- | --- | --- | --- | --- |
| **Fitness to a canonical PES definition:** calculated as the sum of |  |  |  | | |  |  | |
| Transfer directness | [1:4] | Low; Medium; High; Very-high | | | | Muradian et al. (2010) | | |
| User voluntary | [1:2] | Some; All | | | | Voluntariness user side (Wunder 2005) | | |
| Provider voluntary | [1:2] | Some All | |  | | Voluntariness demand side (Wunder 2005) | | |
| ES definition: land use - ES link | [1:3] | Assumed; Used by transferred research; Locally proved | | | | Wunder (2005) criteria (b) | | |
|  |  |  | | |  |  | | |
| Conditionality calculated as the product of: |  |  | | |  | Wunder (2005) criteria (e) | |  |
| (i) Monitoring | [1:3] | Land use; Environmental service; Both | | | |  | | |
| (ii) Enforcement | [1:3] | No enforcement; Weakly applied; Applied | | | |  |  | |
