# Supplementary material for: Global Patterns in the Implementation of Payments for Environmental Services
Source: PLoS One. 2016 Mar 3;11(3):e0149847. doi: 10.1371/journal.pone.0149847 (PMC4777491; doi:10.1371/journal.pone.0149847)
Supplement: S3 Table — (DOCX) [file pone.0149847.s003.docx]

**S3 Table. Additionality assessment evidence.**

| **PES scheme name and country** | **References ^a^** | **Environmental additionality assessment method and data** | **Reported findings^b^** | **Additionality assessment**  **reliability^c^** |
| --- | --- | --- | --- | --- |
| Bol-01 Los Negros, Bolivia | Asquith et al. (2008) | **Qualitative assessment:** PES implementation and operational description.  **Additionality data:** Qualitative deforestation risk assessment. | **NS:** The authors state that additionality is probably low based on the fact that low-threat areas are enrolled. | Suggestive qualitative evidence: Very low. |
| Eq-03 Pimampiro, Ecuador | Wunder and Alban (2008) | **Qualitative assessment:** PES implementation and No baseline but observed land-use patterns indicate a re-vegetation in forest areas. operational description.  **Additionality data:** Forest cover. | **P:** After implementation of the PES scheme local technicians observed an increase in native vegetation in crop and pasture lands. | Suggestive qualitative evidence: Very low. |
| Eq-04 PROFAFOR, Ecuador | Wunder and Alban (2008) | **Qualitative assessment:** PES implementation and operational description. Baseline and reforestation monitoring at farm level. Comparison with national reforestation programs.  **Additionality data:** Forest cover. | **P:** PROFAFOR reforestation plots show a good reforestation rate and have reached higher surfaces than national reforestation programs. | Quantitative causality evidence: Strong. |
| Fra-1 Vittel, France | Perrot-Maitre (2006) | **Quantitative assessment:** PES implementation and operational description and evolution of farm systems.  **Additionality data:** Link between land-use and water chemical quality. | **P:** Payments and agricultural conversion were adopted by farms following hydro-chemical modelling. | Quantitative causality evidence: Strong. |
| Chi-01 Sloping Land Conversion, China | Bennett (2008); Uchida et al. (2007); Xu et al. (2010); Song et al. (2014) | **Quantitative assessment:** PES econometric assessment on land-use change and total income.  **Additionality data:** Cropping outside program areas and tree survival rate. | **P:** A series of studies show that without payments, farmers would have not retired their lands. | Quantitative causality evidence: Strong. |
| Mex-01 Payments for Hydrological Environmental Services (PSAH), Mexico | Alix-Garcia et al. (2012); Alix-Garcia et al. (2014). | **Impact evaluation assessment:** House-hold national level panel data compared using control vs intervention co-variate matching.  **Additionality data:** Forest cover. | **P:** A comparison of forest cover across time between program beneficiaries and rejected applicants using coarse-resolution satellite data suggests that the program has significantly reduced forest loss compared to what would have been expected in the absence of the program in between 5% and 10% depending on spatial heterogeneity. | Quantitative impact evaluation evidence: Very strong. |
| US-01 Conservation Reserve Program (CRP), USA | Lubowski et al. (2003) | **Quantitative assessment:** National wide modelling of land-use change determinants at farm level.  **Additionality data:** Land-use change model. | **P:** Land-use change models predicted that only 15% of total enrolled area would have been conserved anyway. | Quantitative causality evidence: Strong. |
| US-02 Environmental Quality Incentives Program (EQIP), USA | Claassen and Duquette (2012) | **Impact evaluation assessment:** National analysis based on a statistical representative sample on control and program participant house-holds. Propensity score matching to compare participants and non-participants (control) farms.  **Additionality data:** Adoption level of nutrient management, pest management, conservation tillage, soil conservation and buffer practices. | **P:** The authors fond high levels of additionality across the five types of conservation payment types, suggesting that these programs are effective in producing environment gains that would not have occurred without payment incentives | Quantitative impact evaluation evidence: Very strong. |
| UK-01 Environmentally Sensitive Area (ESA) , United Kingdom | Dobbs and Pretty (2008); Boatman et al. (2008). | **Qualitative assessment:** PES implementation and operational description.  **Additionality data:** Aggregated evidence on biodiversity assessment studies. | **NS:** Monetary incentives were adequate to arrest intensification in more marginal areas —where it might not have been profitable to increase the level of intensification anyway — but not adequate to slow or reverse intensification in more productive arable areas | Consistent qualitative evidence: Low. |
| UK-02 Countryside Stewardship Scheme (CSS), United Kingdom | Dobbs and Pretty (2008); Boatman et al. (2008) | **Qualitative assessment:** PES implementation and operational description.  **Additionality data:** Aggregated evidence on biodiversity assessment studies. | **NS:** Overall, most of the UK ESA schemes made positive contributions to ‘greening the edges’ of farming with consequent benefits for habitats and wildlife, but had limited impacts on arable areas and did not succeed in fundamentally changing the sustainability of the crop and livestock operations | Consistent qualitative evidence: Low. |
| Ger-01 Northeim model project, Germany | Klimeka et al. (2008) | **Quantitative assessment:** Implementation, description and statistical analysis of a pilot PES scheme based on an auction system. Monitoring of contracts.  **Additionality data:** Change in ecological points. | **P:** Project implementers verified whether the contracted farmers complied with the statuary requirements of ecological goods, through on the-spot inspections by scientific researchers at the end of the contract period | Quantitative causality evidence: Strong. |
| Zim-01 CAMPFIRE, Zimbabwe | Frost and Bond (2008) | **Qualitative assessment:** Qualitative description of the design and implementation of the program.  **Additionality data:** Long term land-use change and biodiversity monitoring but no further econometric analysis. | **P:** While the total number of elephants has remained more or less constant, there has been a marked shift in distribution away from areas of human habitation. | Consistent qualitative evidence: Low. |
| Co-01 CIPAV-Río La Vieja, Colombia | Zapata et al. (2007); Pagiola et al. (2010) | **Quantitative assessment:** Detailed program design and description combined with house-hold level interviews to all participants.  **Additionality data:** Before and after data on land-use change in participants. | **P:** Before and after qualitative studies show that both poor and rich farmers adopted improved biodiversity agricultural techniques. | Quantitative causality evidence: Strong. |
| Eq-01 Chachis, Ecuador | Wendland and Suárez (2009); Speiser et al. (2009) | No additionality assessment in reviewed studies. | **No evidence:** No evidence although low deforestation threat | No evidence |
| Co-02 Chaina, Colombia | Borda et al. (2009); Rocio-Moreno et al. (2012) | **Quantitative assessment:** Program design and implementation. Soil and Water Assessment Tool (SWAT) was implemented to target areas with high risk of erosion.  **Additionality data:** Erosion baseline and sediment flow during program implementation. | **P:** The ex-post scenario was generated to predict land-use change impacts on soil erosion. After the implementation of the scheme was observed a 33.9% decrease on the production of sediments. | Quantitative causality evidence: Strong. |
| Co-3 Procuenca, Chinchina, Colombia | Cardona-Calle (2008); TUV (2010) | **Qualitative assessment:** PES design and implementation combined with long term socio-economic, land-use and tree health monitoring.  **Additionality data:** Reforested are and tree mortality rate. | **P:** Until 2007, 18 proposals had been accepted with a total reforested land of 2,000ha. | Crosschecked qualitative evidence: Average. |
| Eq-05 Celica, Ecuador | Yaguache-Ordóñez (2013) | **Qualitative assessment:** PES design and implementation qualitative description.  **Additionality data:** Improved land protection status. | **P:** The Celica enterprise bought 17% of lands with hydrological interest and hired a guard to monitor compliance | Suggestive qualitative evidence: Very low. |
| Eq-06 Chaco, Ecuador | Valencia (2010) | **Qualitative assessment:** PES design and implementation qualitative description.  **Additionality data:** Improved land protection status. | **P:** The program has helped to recover 700ha of natural forests. The Municipality of El Chaco has programmed a second phase to recover an additional 2,100 ha of forests | Suggestive qualitative evidence: Very low. |
| SA-01 Richtersveld, Sudafrica | Robinson (1998) | **Qualitative assessment:** PES design and implementation qualitative description.  **Additionality data:** Improved land protection status. | **P:** The protected areas authorities agreed that pastoralists who were prepared to voluntary reduce grazing pressure upon stock units in the national park would be given compensatory land on which to graze equivalent amount of stock units. | Suggestive qualitative evidence: Very low. |
| Ken-01 Kitengela, Kenya | USAID (2012) | **Qualitative assessment:** PES design and implementation qualitative description.  **Additionality data:** Improved land protection status and monitoring. | **P:** Community members are policing the boundaries of the PES areas. In addition, they are apprising landowners of agriculture limits and zoning rules. | Suggestive qualitative evidence: Very low. |
| Mad-01 Menabe, Madagascar | Sommerville et al. (2010) | **Quantitative assessment:** PES design and implementation qualitative description. House-hold and biodiversity surveys.  **Additionality data:** Biodiversity, hunting and monitoring data across time. | **P:** The authors report behavioral changes caused by fear of being caught and punished due to increased monitoring. | Quantitative causality links: Strong. |
| Tan-02 Sea turtle nest, Tanzania | Ferraro (2007) | **Quantitative assessment:** PES design and implementation qualitative description. Quantitative record of poaching rates and monitoring effort combined with ethnographic interviews.  **Additionality data:** Sea turtle before and after poaching rates. | **P:** Poaching rates were 100% prior to 2001. The introduction of salaried monitors in 2001 corresponds with a decline in the poaching rate to about 50%. With the implementation of the performance payment scheme, the poaching rate decreased to 3% in 2002, 2% in 2003 and less than 1% in 2004. | Quantitative causality links: Strong. |
| Cam-01 Bird watch & ecotourism, Cambodia | Clements et al. (2008) ; Clements et al. (2010) | **Qualitative assessment:** PES design and implementation qualitative description. Quantitative record of evolution in bird populations combined with ethnographic interviews.  **Additionality data:** Bird population trends. | **P:** The population of White shouldered Ibis has increased from one nest and a single pair in 2002 to at least six nests and 23 individuals in August 2008. Besides, local people are starting to enforce the land-use plan regulations, by refusing to accept in-migrants and controlling where new forest is cleared. | Crosschecked qualitative evidence: Average. |
| Cam-02 Bird nest protection, Cambodia | Clements et al. (2013) | **Impact evaluation assessment:** Biodiversity monitoring from 2009 to 2011 of a total of 600 nests, protected and non-protected altogether. Short house-hold survey to capture socio-economic benefits and profile of bird nests guards.  **Additionality data:** Bird breeding rates success in control and protected (treated) nests. | **P:** The bird nest program has been extremely successful at protecting nesting sites safeguarding over 1200 nests of globally threatened or near-threatened species since 2002, including 416 nests in 2007–2008. Very few protected nests have been collected by hunters, although it is not uncommon to find unprotected nests that have been collected. | Quantitative impact evaluation evidence: Very strong. |
| Eq-02 SocioBosque, Ecuador | de Koning et al. (2011); Krause et al. (2013) | **Quantitative assessment:** PES design and implementation qualitative description. National deforestation risk zoning.  **Additionality data:** Deforestation risk. | **NS:** Enrolled areas present low deforestation risk because of lack of profitable alternatives or for cultural reasons as in some indigenous communities, thereby making additionality low or inexistent. | Suggestive quantitative evidence: Average. |
| Hon-01 Jesús de Otoro, Honduras | Mejía and Martínez (2006); Kosoy et al. (2007) | **Qualitative assessment:** PES design and implementation qualitative description.  **Additionality data:** Improved land protection status. Water users perceptions of ES quality and quantity. | **P:** The authors state that water users have perceived that water quality and availability have improved after implementation of the PES program. | Consistent qualitative evidence: Low. |
| CR-01 Heredia, Costa Rica | Kosoy et al. (2007); Barrantes and Gámez (2007) | **Qualitative assessment:** PES design and implementation qualitative description.  **Additionality data:** Size of improved land protection status. | **P:** In 2007, almost a third of the total of 1,200 ha of forest described as critical, have been incorporated in the program. No explicit hydrological modelling links protected area with watershed ecosystem services. | Consistent qualitative evidence: Low. |
| Nic-01 San Pedro del Norte, Nicaragua | Kosoy et al. (2007); Obando-Espinoza (2007) | **Qualitative assessment:** PES design and implementation qualitative description.  **Additionality data:** Improved land protection status. Water sources monitoring. | **P:** Water sources have been monitored along the seven years of the project implementation and have experienced a recovery passing from 15 to 19 sources of water with increasing water flows. | Consistent qualitative evidence: Low. |
| Nep-01 Kulekhani, Nepal | Khatri (2009) | **Qualitative assessment:** PES design and implementation qualitative description.  **Additionality data:** Improved land protection status. Road construction in area of the PES program. | **NS:** About 33 percent of total money received under PES mechanisms has been invested in the construction and maintenance of the road that is probably having negative impacts on the siltation of the reservoir. | Suggestive qualitative evidence: Very low. |
| Vie-01 Da Nhim PWS, Dong Nai watershed, Vietnam | Winrock International, (2011) | **Quantitative assessment:** PES zoning targeted areas with high risk of erosion as identified with the Soil Water Assessment Tool (SWAT). Payments were initially aligned with farmers’ opportunity costs.  **Additionality data:** Baseline erosion data and SWAT modelling. | **P:** Soil water assessment tool has allowed identifying areas that cause sediment loads from areas devoted heavily to agriculture. | Quantitative causality evidence: Strong. |
| Vie-02 Son La PWS, Vietnam | Xuan-To et al. (2012) | **Qualitative assessment:** PES design and implementation qualitative description combined with ethnographic surveys.  **Additionality data:** Broad land-use changes, such as resettlement and development infrastructures in PES areas. | **NS:** Many new settlements have been set up for villagers displaced as a consequence of hydropower dam construction as well as two high voltage grids and a road along the provincial border: conservation payments have not avoided deforestation and soil erosion. | Suggestive qualitative evidence: Very low. |
| Indi-01 Oach Kalan - Kuhan mini micro watershed, India | Agarwal et al. (2007) | **Qualitative assessment:** PES design and implementation qualitative description combined with ethnographic surveys. Follow-up of the negotiation process.  **Additionality data:** Improved land protection status. | **P:** PES implementation has created a rule to ban grazing and states the fines to be paid by villagers and migrants  if grazing occurs. | Consistent qualitative evidence: Low. |
| Mex-02 Saltillo, Zapaliname, Mexico | Laurans et al. (2011) | No assessment of environmental additionality. | **No evidence:** Low deforestation threat. Sensitization improved. | No evidence. |
| Tan-01 Simanjiro valley, Tanzania | USAID (2008) | **Qualitative assessment:** PES design and implementation qualitative description combined with ethnographic surveys. Follow-up of the negotiation process.  **Additionality data:** Improved land protection status. | **P:** Since the implementation of the PES scheme villagers have affirmed their boundaries, obtained a Certificate of Village Land and involved government land officers in clarifying the location of the surveyed boundaries. | Consistent qualitative evidence: Low. |
| Nam-01 NRCB management, Namibia | Weaver and Petersen (2008) | **Quantitative assessment:** PES design and implementation qualitative description combined with ethnographic surveys at national level.  **Additionality data:** Wild fauna long term biodiversity monitoring, including before and after the program. | **P:** Community recognition of the value of wildlife has led to a marked reduction in poaching. Besides, the introduction of grassroots wildlife management practices has facilitated a massive recovery of wildlife populations in large communal regions of Namibia. | Suggestive quantitative evidence: Average. |
| US-03 Catskills, NYC, USA | AFD (2010); Grolleau and McCann (2012) | **Quantitative assessment:** PES design and implementation qualitative description. Hydro-chemical modelling linking farming practices with water quality.  **Additionality data:** Land-use change dynamics and improved land protection status. | **P:** In between 1997 and 2008, the city of New York included in the program around 36,800 ha of the Catskills-Delaware watershed. The City now controls 13.5% of the total surface watershed, whereas it controlled only 3.5% in 1996. | Quantitative causal evidence: Strong. |
| Tan-02 Tanzania, PWS | Laurans et al. (2012); Branca et al. (2011) | No assessment of environmental additionality. | **No evidence**: Given the relatively small area of land covered by improved land management practices, it is not yet possible to detect any significant change in water quality indicators downstream, and it is not known how large the intervention area will need to be to demonstrate real impact. | No evidence |
| Nic-02 Silvopastoril, Nicaragua | Pagiola et al. (2007) | **Quantitative assessment:** Farm level analysis before and after implementation of the scheme.  **Additionality data:** Land-use change dynamics including tree density for silvopastoral systems. | **P:** The area of degraded pasture fell by two thirds, while pastures with high tree density increased substantially, as did fodder banks and live fences. On-going monitoring indicates that land-use changes are generating the expected carbon storage and soil conservation services. | Quantitative causality evidence: Strong. |
| CR-03 Silvopastroril, Costa Rica | Ibrahim et al. (2007) | **Quantitative assessment:** Farm level analysis before and after implementation of the scheme.  **Additionality data:** Land-use change dynamics including tree density for silvopastoral systems. | **P:** In between 2003 and 2006, important areas of degraded pastures were converted to improved pastures with trees. | Quantitative causality evidence: Strong. |
| Ind-01 Cidanau watershed PES scheme, Indonesia | Leimona et al. (2010) | No assessment of environmental additionality. | **No evidence:** There is insufficient scientific evidence to judge the impacts of the Cidanau scheme on environmental services since the causal link between changing land-use practices and increasing ES is not known. | No evidence. |
| Ug-01 Trees for Global Benefits Program, Uganda | Ecotrust (2010); German et al. (2010) | **Qualitative assessment:** PES design and implementation qualitative description combined with ethnographic surveys.  **Additionality data:** Perception of improved ES. Tree survival rate. | **P:** Farmers stated improved crop productivity as a result of better land management techniques and they also perceived microclimatic changes that they viewed as favorable, such as cooler temperatures and more regular rainfall. They also believed that air was fresher, due to the presence of forests. In some cases, trees have provided an important windbreak, reducing the negative impact of high winds on homes and banana plantations. | Consistent qualitative evidence: Low. |
| Phi-01 Philippines, No Fire Bonus Scheme | Soriaga and Annawi (2010) | **Qualitative assessment:** PES design and implementation qualitative description combined with ethnographic surveys.  **Additionality data:** Improved land protection status. | **NS:** Municipal-level baseline statistics before the scheme’s implementation were not available to allow comparison with the post-implementation trend, which shows a declining trend in the extent of forest fires after one cycle of implementation." | Suggestive qualitative evidence: Very low. |
| Moz-01 Mozambique, carbono. | Hegde and Bull (2011); Jindal et al. (2012) | **Quantitative assessment:** PES design and implementation qualitative description combined with socio-economic before and after house-hold surveys.  **Additionality data:** Tree survival rate. | **P:** The project had a good impact on restoring above biomass carbon through plantations. | Quantitative causality evidence: Strong. |
| Mex-02 Scolel Té | Scolel'Te (2011) | **Quantitative assessment:** PES design and implementation qualitative description combined with socio-economic before and after house-hold surveys.  **Additionality data:** Tree survival rate. | **P:** Tree survival rate is more than 85%. | Quantitative causality evidence: Strong. |
| Mex-03 Monarch | Honey-Roses et al. (2011) | **Impact evaluation assessment:** PES design and implementation qualitative description combined with long term land-use change and forest cover.  **Additionality data:** Forest cover evolution from 1986 to 2099. | **P:** Conservation measures protected between 200 ha and 710 ha (3–16%) of forest that is high-quality habitat for monarch butterflies, but had a smaller effect on total forest cover, preserving between 0 ha and 200 ha (0–2.5%) of forest with canopy cover above 70%. | Quantitative impact evaluation evidence: Very strong. |
| Ger-02 PWS en Munich | Grolleau and McCann (2012) | **Quantitative assessment:** PES design and implementation qualitative description. Hydro-chemical modelling linking farming practices with water quality.  **Additionality data:** Monitoring in farms’ agricultural systems. | **P:** The switch to organic farming was self-enforcing thanks to at least two mechanisms, namely, a mix of financial incentives until organic farming became more profitable than conventional farming, and strong involvement of the city in purchasing and promoting organic products from the targeted area as well as acquisition of specific skills and reputation. | Quantitative causality evidence: Strong. |
| In-01 Sumberjaya AF conservation auction | Leimona et al. (2009) | **Quantitative assessment:** Pilot PES based on an auction system to implement soil conservation techniques.  **Additionality data:** Monitoring the implementation of soil conservation techniques. | **P:** More than 50% of farmers accomplished their contracts. | Suggestive quantitative: Average. |
| Ken-02 Reforestation conservation auction in W Kenya | Wünscher et al. (2013) | **Quantitative assessment:** Pilot PES based on an auction system to implement reforestation practices in bared soil.  **Additionality data:** Tree survival rate. | **P:** The program site has shown a seedling survival of 87%. | Quantitative causality evidence: Strong. |
| Co-04 Fúquene, Colombia | Quintero and Otero (2006) | **Quantitative assessment:** PES design and implementation qualitative description. Hydro-chemical modelling linking farming practices with water quality.  **Additionality data:** Monitoring in farms’ agricultural systems. | **P:** During 2004, the Andean Watersheds regional project helped implement a total credit amount of 200,000 USD for 39 small producers that introduced green fertilizers for a total size of 85.25 ha. From these credits, 99% of the money has been recovered and 97% of the contracted surface has been kept for the application of green fertilizers during following crops. | Quantitative causality evidence: Strong. |
| Bol-02 Noel Kempff Mercado REDD+ project | Robertson and Wunder (2005); Couto-Pereira (2010) | **Qualitative assessment:** PES design and implementation qualitative description combined with stakeholder analysis at national level.  **Additionality data:** Improved land protection status. | **NS:** The REDD+ zone was placed in areas where logging concessions had already planned to leave and of very difficult access. | Suggestive qualitative evidence: Very low. |
| CR-02 Payments for Environmental Services (PSA), Costa Rica | Sanchez-Azofeifa et al. (2007) ; Pfaff et al. (2008); Arriagada et al. (2012) | **Impact evaluation assessment:** Combined sampling of data from remote sensing and household  surveys, compared through, difference-in-difference matching estimators, and tests of sensitivity to unobservable heterogeneity.  **Additionality data:** Forest cover evolution. | **NS:** Mixed evidence, ranging from no additionality (Sanchez-Azofeifa et al. 2007; Pfaff et al. 2008) to medium additionality (Arriagada et al 2012). Large forested tracts owned by absentee landlords and with steeper slopes have a higher probability of enrolment in a PES program in Costa Rica. | Quantitative impact evaluation evidence: Very strong. |
| Co-05 CIPAV- La Salvajina & Munchique, Colombia | CIPAV (2007) | **Qualitative assessment:** PES design and implementation qualitative description combined with ethnographic data.  **Additionality data:** Improved land protection status and tree planting to prevent soil erosion. | **P:** The implementation of the scheme has allowed for the maintenance and more frequent implementation of local collaborative Exchange of work force called MINGAS. | Consistent qualitative evidence: Low. |
| Mad-02 Makira WCS Madagascar | Brimont et. al. (2015) | **Qualitative assessment:** PES design and implementation qualitative description combined with house-hold surveys.  **Additionality data:** Improved land protection status including earth-satellite monitoring. | **NS:** The REDD+ scheme is reducing deforestation pressure in the areas most vulnerable to climatic risk that had already very low deforestation risk. | Consistent qualitative evidence: Low. |
| Bol-03 Landrace conserv payments Bolivia & Peru | Narloch et al. (2011) | **Quantitative assessment:** Pilot PES based on an auction system to induce trade-off outcomes in terms of payments repartition and biodiversity protection.  **Additionality data:** Diversity and surface of agricultural landraces protected. | **P:** Each of the pilot targeting approaches that were implemented produced a different cost-effectiveness ranking. | Qualitative causality evidence: Strong. |
| Bra-1 Bolsa Floresta - Brasil | Borner et al. (2013) | **Impact evaluation assessment:** Combined sampling of data from remote sensing and spatially explicit data from deforestation fines compared through, difference-in-difference matching estimators.  **Additionality data:** Forest cover evolution. | **NS:** The authors find that the Bolsa Floresta PES group of protected areas is doing slightly better than their counterparts not receiving the program in curbing deforestation. | Quantitative impact evaluation evidence: Very strong. |

^a^: See full references cites in Supporting Information S2.

^b^:NS: Not significant; P: Positive.

^c^: Score for criteria for additionality assessment: Statistical evidence yes/no; quality of statistical protocol; qualitative evidence yes/no; quality of qualitative evidence; alternative studies reinforcing or contradicting the assessment
