# Supplementary material for: Global Patterns in the Implementation of Payments for Environmental Services
Source: PLoS One. 2016 Mar 3;11(3):e0149847. doi: 10.1371/journal.pone.0149847 (PMC4777491; doi:10.1371/journal.pone.0149847)
Supplement: S4 Table — (DOCX) [file pone.0149847.s004.docx]

**S4 Table. Additionality assessment summary.**

| Level of precision | Number of cases | Type of evidence used |
| --- | --- | --- |
| Weak | 10 | Qualitative descriptions of PES environmental outcomes with suggestive evidence. |
| Fragile | 11 | Specific qualitative study or section of a study where consistent qualitative evidence on environmental additionality is presented |
| Medium | 5 | (i) Qualitative evidence crosschecked by different studies;  Or  (ii) Suggestive quantitative evidence based on trends with no inference or causality assessments. |
| Strong | 19 | Quantitative causal assessment based on modelling and representative statistical sampling. |
| Rigorous | 6 | Impact evaluation based on before-after-control-intervention techniques or matching control vs. treatment units. |
