# Supplementary material for: Global Patterns in the Implementation of Payments for Environmental Services
Source: PLoS One. 2016 Mar 3;11(3):e0149847. doi: 10.1371/journal.pone.0149847 (PMC4777491; doi:10.1371/journal.pone.0149847)
Supplement: S5 Table — (DOCX) [file pone.0149847.s005.docx]

**S5 Table. ANOVA and t-test means differences for payments amounts and size of PES programs, by sector and targeted ES.**

The median and standard error variations of the PES scheme size in hectares and total payments in USD/ha/yr show important differences (Figure 2 in the manuscript). Supporting table 4 explores in detail such differences using ANOVA test. Public sector payments score higher than private commercial and non-profit private schemes. Water PES present the highest payments and the lowest surfaces. Biodiversity payments score the lowest values (in USD/ha/yr) and the highest surfaces.

|  | Means differences | | |  |
| --- | --- | --- | --- | --- |
|  | Log10 Payment (USD/ha/yr) |  | Log10 Size (Ha) |  |
|  |  |  |  |  |
| Pr-P | -0.53 | * | -0.25 |  |
| NP-P | -0.90 | ** | -0.05 |  |
| Pr-NP | 0.37 |  | -0.19 |  |
| W-Bio | 1.55 | *** | -1.12 | * |
| W-C | 0.63 | * | -1.16 | * |
| W-MA | 0.32 |  | -1.78 | ** |
| Bio-C | -0.91 | ** | -0.04 |  |
| Bio-MA | -1.22 | *** | -0.66 |  |
| C-MA | -0.31 |  | -0.62 |  |

* p<0.1; **p<0.05; ***p<0.001

P: Public; Pr: Private commercial; NP: Private non-profit; W: Water; Bio: Biodiversity; C: Carbon; MA: multi-functional agriculture.
