# Supplementary material for: Global Patterns in the Implementation of Payments for Environmental Services
Source: PLoS One. 2016 Mar 3;11(3):e0149847. doi: 10.1371/journal.pone.0149847 (PMC4777491; doi:10.1371/journal.pone.0149847)
Supplement: S6 Table — (DOCX) [file pone.0149847.s006.docx]

**S6 Table. Rotated component loadings from a two-dimensional CatPCA on 26 PES variables, with all variables analysed nominally excepted market composition and FitCanPES level.**

|  | Component |  | Model summary | | | | |
| --- | --- | --- | --- | --- | --- | --- | --- |
|  | 1 | 2 |  | Dimension | Cronbach's Alpha | Variance Accounted For | |
| Water | -0,06 | **0,52** |  |  |  | Total (Eigenvalue) | % of Variance |
| Biodiver | **0,8** | **-1,14** |  | 1 | 0,839 | 3,762 | 47,024 |
| Carbon | **0,63** | **0,44** |  | 2 | 0,799 | 3,321 | 41,514 |
| Agricult | **-1,18** | -0,02 |  | Total | ,869^a^ | 4,171^c^ | 52,133^c^ |
| Forest | -0,14 | -0,06 |  |  |  |  |  |
| Farmland | -0,2 | **0,4** |  |  |  |  |  |
| Semi-ari | **1,14** | **-1,02** |  |  |  |  |  |
| FitCanPes Low | **-0,63** | 0,02 |  |  |  |  |  |
| FitCanPes Medium | **-0,42** | -0,34 |  |  |  |  |  |
| FitCanPes High | **0,65** | **-0,79** |  |  |  |  |  |
| FitCanPes Very-high | **1,05** | **1,06** |  |  |  |  |  |
| Upfront Public | **-0,45** | 0,28 |  |  |  |  |  |
| Upfront Non-prof | **0,65** | **-1,32** |  |  |  |  |  |
| Upfront Private | **1,5** | **0,89** |  |  |  |  |  |
| Recurrent Public | **-0,73** | 0,17 |  |  |  |  |  |
| Recurrent Non-prof | **0,68** | **-1,31** |  |  |  |  |  |
| Recurrent Private | **1,2** | **0,87** |  |  |  |  |  |
| Payments Public | **-0,8** | 0,13 |  |  |  |  |  |
| Payments Non-prof | **0,71** | **-1,98** |  |  |  |  |  |
| Payments Private | **1,2** | **0,73** |  |  |  |  |  |
| Monopsone | -0,36 | 0,01 |  |  |  |  |  |
| Oligopsone | **0,73** | **-1,14** |  |  |  |  |  |
| Club | 0,02 | 0,65 |  |  |  |  |  |
| Market | **1,85** | 0,28 |  |  |  |  |  |
| Source user = No | **-0,56** | **-0,61** |  |  |  |  |  |
| Source users = Yes | **0,5** | **0,55** |  |  |  |  |  |

*Note. Loadings higher than .40 are shown in bold.*

Component loadings with weights above 0.4 are considered explicative of its respective dimension (Linting and van der Kooij, 2014).

**Additional references:**

Linting M, van der Kooij A. 2012. Nonlinear Principal Components Analysis With CATPCA: A

Tutorial. Journal of Personality Assessment, 94(1): 12-25.
