# Supplementary material for: Global Patterns in the Implementation of Payments for Environmental Services
Source: PLoS One. 2016 Mar 3;11(3):e0149847. doi: 10.1371/journal.pone.0149847 (PMC4777491; doi:10.1371/journal.pone.0149847)
Supplement: S7 Table — (DOCX) [file pone.0149847.s007.docx]

S7 Table. Pearson correlation levels and significance between the predictor variables of the bivariate additionality model.

| **N=51** |  | Activity paid | Diversification of payments | Spatial targeting | Conditionality | Additionality assessment precision | Time (years of PES) |
| --- | --- | --- | --- | --- | --- | --- | --- |
| Activity paid | R^2^ | 1 |  |  |  |  |  |
|  | Sig. (2-tailed) |  |  |  |  |  |  |
| Diversification of payments | R^2^ | 0,18 | 1 |  |  |  |  |
|  | Sig. (2-tailed) | 0,21 |  |  |  |  |  |
| Spatial targeting | R^2^ | 0,07 | -0,07 | 1 |  |  |  |
|  | Sig. (2-tailed) | 0,64 | 0,64 |  |  |  |  |
| Conditionality | R^2^ | 0,14 | 0,07 | 0,15 | 1 |  |  |
|  | Sig. (2-tailed) | 0,32 | 0,65 | 0,30 |  |  |  |
| Additionality assessment precision | R^2^ | 0,18 | ,280^*^ | -0,02 | 0,26 | 1 |  |
|  | Sig. (2-tailed) | 0,21 | 0,05 | 0,88 | 0,07 |  |  |
| Time (years of PES) | R^2^ | ,313^*^ | 0,14 | 0,11 | 0,10 | -0,01 | 1 |
|  | Sig. (2-tailed) | 0,03 | 0,33 | 0,44 | 0,50 | 0,96 |  |
| Sector=Private profit | R^2^ | 0,20 | 0,04 | -0,05 | ,452^**^ | -0,10 | 0,08 |
|  | Sig. (2-tailed) | 0,16 | 0,80 | 0,71 | 0,00 | 0,50 | 0,58 |
| Sector=Private non-profit | R^2^ | -0,05 | -0,10 | ,391^**^ | 0,06 | 0,17 | -0,08 |
|  | Sig. (2-tailed) | 0,75 | 0,49 | 0,01 | 0,68 | 0,24 | 0,60 |
| Sector=Public | R^2^ | -0,15 | 0,04 | -0,22 | -,451^**^ | -0,03 | -0,02 |
|  | Sig. (2-tailed) | 0,29 | 0,81 | 0,11 | 0,00 | 0,84 | 0,89 |
