# Supplementary material for: Global Patterns in the Implementation of Payments for Environmental Services
Source: PLoS One. 2016 Mar 3;11(3):e0149847. doi: 10.1371/journal.pone.0149847 (PMC4777491; doi:10.1371/journal.pone.0149847)
Supplement: S8 Table — (DOCX) [file pone.0149847.s008.docx]

S8 Table. Collinearity analysis for the predictor variables of the bivariate additionality model.

|  |  | Model 1 | | Model 2 | | Model 3 | | Model 4 | |
| --- | --- | --- | --- | --- | --- | --- | --- | --- | --- |
| Dependent variable | | Diversification of payments | | Spatial targeting | | Conditionality | | Additionality assessment precision | |
|  |  | Tolerance | VIF | Tolerance | VIF | Tolerance | VIF | Tolerance | VIF |
| Diversification of payments | |  |  | 0,87 | 1,14 | 0,88 | 1,14 | 0,95 | 1,06 |
| Spatial targeting | | 0,79 | 1,26 |  |  | 0,81 | 1,23 | 0,81 | 1,23 |
| Conditionality | | 0,67 | 1,50 | 0,68 | 1,47 |  |  | 0,75 | 1,34 |
| Additionality assessment precision | | 0,81 | 1,23 | 0,77 | 1,30 | 0,84 | 1,19 |  |  |
| Time (years of PES) | | 0,88 | 1,14 | 0,87 | 1,14 | 0,87 | 1,15 | 0,87 | 1,14 |
| Sector=Private profit | | 0,66 | 1,51 | NA | NA | 0,89 | 1,12 | 0,70 | 1,42 |
| Sector=Private non-profit | | 0,76 | 1,32 | 0,67 | 1,50 | 0,75 | 1,33 | 0,77 | 1,30 |
| Sector=Public | | NA | NA | 0,55 | 1,83 | NA | NA | NA | NA |
| Activity paid | | 0,83 | 1,21 | 0,82 | 1,21 | 0,82 | 1,22 | 0,85 | 1,18 |

|  |  | Model 5 | | Model 6 | | Model 7 | | Model 8 | | Model 9 | |
| --- | --- | --- | --- | --- | --- | --- | --- | --- | --- | --- | --- |
| Dependent variable | | Time (years of PES) | | Sector=Private profit | | Sector=Private non-profit | | Sector=Private public | | Activity paid | |
|  |  | Tolerance | VIF | Tolerance | VIF | Tolerance | VIF | Tolerance | VIF | Tolerance | VIF |
| Diversification of payments | | 0,88 | 1,13 | 0,87 | 1,14 | 0,87 | 1,14 | 0,87 | 1,14 | 0,88 | 1,14 |
| Spatial targeting | | 0,80 | 1,24 | 0,79 | 1,26 | 0,79 | 1,26 | 0,79 | 1,26 | 0,80 | 1,25 |
| Conditionality | | 0,67 | 1,50 | 0,66 | 1,50 | 0,66 | 1,50 | 0,66 | 1,50 | 0,67 | 1,50 |
| Additionality assessment precision | | 0,76 | 1,32 | 0,75 | 1,33 | 0,75 | 1,33 | 0,75 | 1,33 | 0,78 | 1,28 |
| Time (years of PES) | |  |  | 0,87 | 1,15 | 0,87 | 1,15 | 0,87 | 1,15 | 0,95 | 1,06 |
| Sector=Private profit | | 0,66 | 1,51 |  |  | 0,34 | 2,95 | 0,66 | 1,51 | NA | NA |
| Sector=Private non-profit | | 0,75 | 1,33 | 0,57 | 1,76 |  |  | 0,75 | 1,34 | 0,58 | 1,71 |
| Sector=Public | | NA | NA | 0,54 | 1,84 | 0,37 | 2,74 |  |  | 0,56 | 1,77 |
| Activity paid | | 0,89 | 1,12 | 0,82 | 1,22 | 0,82 | 1,22 | 0,82 | 1,22 |  |  |

We check for collinearity in the predictor variables of the bivariate logistic additionality model taking into account the existence of a VIF > 3 and a tolerance level < 0.2 in between the predictor variables. The "tolerance" captures the percent of variance in the predictor that cannot be accounted for by the other predictors. Very small values indicate that a predictor is redundant so that values that are less than .10 may merit further collinearity checks. The VIF, which stands for variance inflation factor, is (1 / tolerance). Values greater than 3 merit further investigation. We find no evidence of collinearity.
