# Supplementary material for: Global Patterns in the Implementation of Payments for Environmental Services
Source: PLoS One. 2016 Mar 3;11(3):e0149847. doi: 10.1371/journal.pone.0149847 (PMC4777491; doi:10.1371/journal.pone.0149847)
Supplement: S9 Table — (DOCX) [file pone.0149847.s009.docx]

S9 Table. Logistic regression models with cut-off level set at 0.25, 0.50 and 0.75.

|  | Model | % Predicted | Nagelkerke R2 | H-L test p | -2 log likelihood |  |
| --- | --- | --- | --- | --- | --- | --- |
|  |  |  |  |  |  |  |
| Cut-off 0.25 | M31 | 92.2 | 0.79 | 0.55 | 18.00 |  |
| Cut-off 0.75 | M33 | 90.2 | 0.79 | 0.55 | 18.00 |  |
| Cut-off 0.50 | M34 | 94.1 | 0.79 | 0.55 | 18.00 |  |

We run the bivariate logistic model at three different probability cut-off levels (0.25, 0.50 and 0.75) to test for the influence in the model of an environmental additionality decided ex-ante. The best prediction is obtained with the equal-probability assumption of 0.50. We find no changes in model descriptors. The highest accuracy is obtained with the equal-probability assumption of 0.50.
